# Impact of different training modalities on anthropometric outcomes in patients with obesity: A systematic review and network meta‐analysis

**DOI:** 10.1111/obr.13218

**Published:** 2021-02-23

**Authors:** Jakub Morze, Gerta Rücker, Anna Danielewicz, Katarzyna Przybyłowicz, Manuela Neuenschwander, Sabrina Schlesinger, Lukas Schwingshackl

**Affiliations:** ^1^ Department of Cardiology and Internal Diseases University of Warmia and Mazury Olsztyn Poland; ^2^ Department of Human Nutrition University of Warmia and Mazury Olsztyn Poland; ^3^ Institute of Medical Biometry and Statistics, Medical Center ‐ University of Freiburg, Faculty of Medicine University of Freiburg Freiburg Germany; ^4^ Institute for Biometrics and Epidemiology, German Diabetes Center (DDZ), Leibniz Center for Diabetes Research Heinrich Heine University Düsseldorf Düsseldorf Germany; ^5^ Institute for Evidence in Medicine, Medical Center ‐ University of Freiburg, Faculty of Medicine University of Freiburg Freiburg Germany

**Keywords:** aerobic exercise, GRADE, network meta‐analysis, resistance exercise

## Abstract

Obesity management guidelines consistently advise aerobic training for weight loss, whereas recommendations for other training modalities are sparse. This systematic review and network meta‐analysis (NMA) aimed to compare the long‐term effects of different training modalities on anthropometric outcomes in patients with obesity. MEDLINE, Cochrane CENTRAL, and Web of Science were searched to identify the following: (1) randomized controlled trials (RCTs); (2) conducted in adults with a mean body mass index (BMI) ≥30 kg/m^2^; (3) comparing aerobic, resistance, combined, or high‐intensity interval training head‐to‐head or to control for ≥6 months; and (4) reporting changes in body weight (BW), BMI, waist circumference (WC), fat mass (FM), or fat‐free mass (FFM). Random‐effects NMA models were fitted in a frequentist approach. GRADE framework was used to assess certainty of evidence. Thirty‐two RCTs with 4774 participants with obesity were included in this review. Aerobic training was ranked as best for improving BW, BMI, and WC and combined training for improving FM, as well as equally with resistance training most effective for improving FFM. Low to moderate certainty of evidence supports use of aerobic training to improve anthropometric outcomes in obesity, and its combination with resistance training provides additional benefit for reducing FM and increasing FFM.

**Abbreviations**
AET: aerobic training
BMI: body mass index
BW: body weight
CI: confidence interval
CoE: certainty of evidence
CONT: control
CT: combined training
FFM: fat‐free mass
FM: fat mass
HIIT: high‐intensity interval training
MD: mean difference
MI: minimal intervention
NMA: network meta‐analysis
RCT: randomized controlled trial
RT: resistance training
WC: waist circumference

## 1 INTRODUCTION

The World Health Organization defines obesity as a body mass index (BMI) of 30 kg/m^2^ or more.^1^ Over the past 40 years, the worldwide number of adults with obesity increased more than sixfold, becoming a global epidemic.^2^ Excessive body fat accumulation greatly increases the risk of mortality, cardiovascular disease, diabetes, cancer, and other chronic diseases.^3^, ^4^, ^5^ Considering the burden of health‐related consequences, effective management of obesity seems to be of great public importance.

Increasing physical activity and limiting energy intake to create a negative energy balance are standard methods to promote weight loss. There is a joint agreement between guidelines that advising moderate‐intensity aerobic exercise of at least 150 min/week is sufficient to assure clinically meaningful weight loss.^6^, ^7^, ^8^, ^9^, ^10^, ^11^ This is supported by previous meta‐analyses showing that aerobic exercise reduces body weight (BW), waist circumference (WC), and subcutaneous and visceral adiposity.^12^, ^13^, ^14^, ^15^

However, there are conflicting views on the use of resistance exercise alone or in combination with aerobic exercise for weight loss. In their position paper, the American College of Sports Medicine states that resistance training seems non‐effective for weight loss.^6^ The 2016 guidelines from the American Association of Clinical Endocrinology/American College of Endocrinology recommended the addition of resistance training to weight loss programs to promote more significant fat reduction while preserving fat‐free mass.^7^ Recently, *Villareal* and colleagues showed that adding resistance training to weight management programs with a hypocaloric diet for six months led to similar weight reduction as adding aerobic or combined training.^16^

High‐intensity interval training (HIIT) has emerged as an alternative to aerobic and resistance training, which is intermittent high‐intensity aerobic exercise interspersed by periods of rest and recovery.^17^ The increasing popularity of HIIT is because it shows the potential of being as effective as continuous aerobic training while requiring less time per session.^18^, ^19^

Given the different exercise modalities available, the question remains, which of them is the most effective for weight loss. Network meta‐analysis (NMA) extends the idea of a pairwise meta‐analysis by providing simultaneous comparisons of multiple treatments using direct and indirect evidence.^20^ Previous NMA comparing the effects of aerobic, resistance, and combined training on body composition included only short‐term (<6 months), head‐to‐head studies, conducted in participants with both overweight and obesity.^21^ Results of long‐term exercise trials can differ substantially from short‐term ones because of time‐decreasing adherence to training protocols or compensation by increased energy intake.^22^, ^23^ Moreover, since the publication of the earlier review, several new trials have been published.^16^, ^24^, ^25^, ^26^, ^27^

Therefore, we aimed to conduct a systematic review with a network meta‐analysis to compare different training modalities and rank their long‐term effects on anthropometric outcomes (BW, BMI, WC, fat mass [FM], and fat‐free mass [FFM]) in patients with obesity. Moreover, we assessed the certainty of available evidence to inform decision‐making regarding public health recommendations.

## 2 METHODS

This systematic review has a predefined protocol published in the PROSPERO International Prospective Register of Systematic Reviews (CRD42020178548). We followed the PRISMA Extension for Network Meta‐analyses (PRISMA‐NMA) checklist when reporting methods and results of the current review.^28^

### 2.1 Study search

We conducted a comprehensive search of three electronic databases, including MEDLINE® (OvidSP), the Cochrane Central Register of Controlled Trials (CENTRAL), and Web of Science (search period from inception to September 7, 2020) (Table S1). For study searching, we did not use any restrictions or filters. Additionally, we searched the WHO International Clinical Trials Registry Platform and ClinicalTrials.gov to identify potential ongoing trials. We screened reference lists from retrieved articles, systematic reviews, and meta‐analyses to identify eligible studies not covered by database search. A study search was completed by two authors (J.M. and L.S.).

### 2.2 Eligibility criteria

Two authors (J.M. and A.D.) independently screened database search results by titles and abstracts and later assessed the eligibility of identified full‐text articles. Any disagreements at this step were resolved by discussion with a third author (L.S.) We have included studies in the review if they fulfilled all of the following criteria:

- Parallel or cross‐over randomized controlled trials (RCTs).
- Conducted in adults (mean or median age ≥18 years), with a mean BMI ≥30 kg/m^2^, as it has been done in previous systematic reviews.^29^ Due to the different definitions of obesity in Asia,^30^ we used a criterion of BMI ≥25 kg/m^2^ for studies conducted in that region. If an intervention arm of an RCT did not meet BMI criteria, we excluded it.
- Compared any type of exercise modality with another or with control or minimal intervention for at least six months (24 weeks). We considered studies that provided a detailed exercise protocol, including information on exercise modality type, session duration and frequency, and exercise intensity, as defined by the American College of Sports Medicine.^31^ We excluded studies where exercise protocols were missing any of the details mentioned above. Moreover, we did not consider exercise programs that solely used subjective exercise intensity (i.e., Borg RPE Scale).^32^ Studies in which some of the arms did not meet the criteria for exercise protocol were included if, after the removal of non‐eligible arms, a comparison of interest could still be extracted. We allowed for the presence of co‐interventions (such as dietary intervention or social support), as long as those were balanced across study arms within an RCT. The following training modalities were considered as eligible for inclusion:
- Aerobic training (AET): that is, treadmill, ergometer cycling, jogging, or walking.
- Resistance training (RT): that is, weight‐lifting machines or strength exercises on major muscle groups.
- Combined aerobic and resistance training (CT).
- HIIT.

The comparison group was defined as control (CONT) or minimal intervention (MI), including no formal exercise program or maintaining usual physical activity; participation in lifestyle education/group meetings, light exercise (i.e., stretching). As our primary aim was to identify exercise trials, assessing MI as a separate treatment might be biased by missing evidence. Therefore, CONT was analyzed together with MI and later split as distinct nodes in sensitivity analyses.^33^

### 2.3 Data extraction

After identification of eligible articles, two reviewers (J.M. and A.D.) extracted the following characteristics: name of the first author, year of publication, study location (country), study design (parallel or cross‐over), study length (months), sample size (number of randomized participants), percentage of female, mean age, mean BMI, available comparisons, specification of the exercise programs (exercise type, duration, frequency, and intensity), presence and description of a balanced co‐intervention, outcomes extracted for the current review, and conflict of interest. In the case of missing information, we made two attempts to contact authors by e‐mail (summary of additional data is available in Table S2). When extracting outcome data, we prioritized the use of adjusted change scores with corresponding standard deviations, followed by change scores. If available, missing change scores were calculated from pre‐ and post‐intervention values using a correlation coefficient of 0.5 according to the formula provided by *Cochrane Handbook*.^34^ Change scores expressed as percentages were converted to crude change scores by multiplying them per pre‐intervention means. A third author (L.S.) verified the extracted data.

### 2.4 Risk of bias assessment

Two pairs of authors (J.M./L.S. and S.S./M.N) assessed the risk of bias of included studies independently using the revised Cochrane risk‐of‐bias tool for randomized trials (RoB 2),^35^ with any disagreements resolved by consensus. The RoB 2 tool consists of five domains: bias arising from the randomization process, bias due to deviations from the intended interventions, bias due to missing outcome data, bias in the measurement of the outcome, and bias in the selection of the reported result. Details of the RoB2 assessment are provided in Table S3. The overall risk of bias for a study was judged as low, some concerns, or high risk.

### 2.5 Statistical analysis

We illustrated the available direct comparisons between distinct exercise modalities using network graphs, separately for BW, BMI, WC, FM, and FFM. The nodes' size is proportional to the sample size available for each exercise modality, and the thickness of the lines is proportional to the number of studies available for specific comparisons.

We calculated mean differences (MD) with 95% confidence intervals (95% CI) between change scores for all available comparisons, which were used as effect measure in further analyses. For each of the outcomes, we have fitted a random‐effects NMA in a frequentist framework.^20^ NMA extends the idea of a classical pairwise meta‐analysis by comparing multiple interventions simultaneously while preserving individual trials' internal randomization.^20^ We have presented results of NMA using forest plots and league tables. In order to produce a relative ranking of exercise modalities effects on the anthropometric outcome, we calculated P‐scores. P‐score is a frequentist analogue of the surface under the cumulative ranking curves available in the Bayesian approach.^36^ The P‐score value ranges between 0 and 1, indicating the intervention ranked as the worst and the best.

To verify the assumption of transitivity, we visually explored the distribution of potential effect modifiers across available direct comparisons. We have considered the following effect modifiers: age, sex, baseline BMI, study location, and length of follow‐up.

We adopted the node‐splitting approach, which separates evidence from a certain comparison into direct and indirect effect to check for the presence of local inconsistency.^37^ Moreover, we created net heat plots using a full design‐by‐treatment model to find designs contributing to the presence of substantial inconsistency.^38^ Design can be defined as the set of treatments compared within a trial.^38^ Additionally, to assess between‐study heterogeneity changes in subgroup/sensitivity analyses, we used overall network τ^2^ and I^2^ statistic with 95% CI.^39^

Subgroup analyses were stratified for age group (<65 years vs. ≥65 years; commonly considered as the age for elderly adults),^40^ sex (male vs. female), baseline BMI (<35 kg/m^2^ vs. ≥35 kg/m^2^; cut‐off point for WHO Class II obesity),^1^ length of follow‐up (<12 months vs. ≥12 months),^41^ and dietary co‐intervention (present vs. absent). Sensitivity analyses were run by excluding studies with a high risk of bias and by splitting control and minimal intervention as separate nodes.

Comparison‐adjusted funnel plots and Egger's linear regression tests were adopted to explore for presence of small‐study effects.^42^

We conducted all analyses in R version 4.0.2 (R Foundation for Statistical Computing, Vienna, Austria), with the use of packages *meta* ^43^ and *netmeta*.^39^

### 2.6 Certainty of evidence

To evaluate the certainty of evidence (CoE) derived from NMA, we implemented the Grading of Recommendations Assessment, Development, and Evaluation (GRADE) approach.^44^ For all outcomes, one author (L.S.) completed the GRADE assessment for each of the direct, indirect, and network estimates. Evaluation of direct estimates included the following GRADE domains: risk of bias, indirectness, inconsistency, and publication bias. According to the GRADE working group's recent suggestion, consideration of imprecision is not necessary when rating the direct and indirect estimates to assess the rating of network estimates.^44^ We used the certainty of direct estimates to inform indirect estimates (the lowest of the ratings of the two direct comparisons forming the most dominant first‐order loop), and eventually, we rated them down in the presence of serious intransitivity. The dominant first‐order loop can be defined as a triangular set of nodes contributing most to a specific indirect estimate. We compared respective ratings for direct and indirect estimates to address the certainty of network estimates (the one with higher certainty was chosen), and we rated them down if incoherence or imprecision were detected.^44^ GRADE classifies the certainty of evidence in one of four levels: high, moderate, low, and very low.

## 3 RESULTS

**FIGURE 1:**
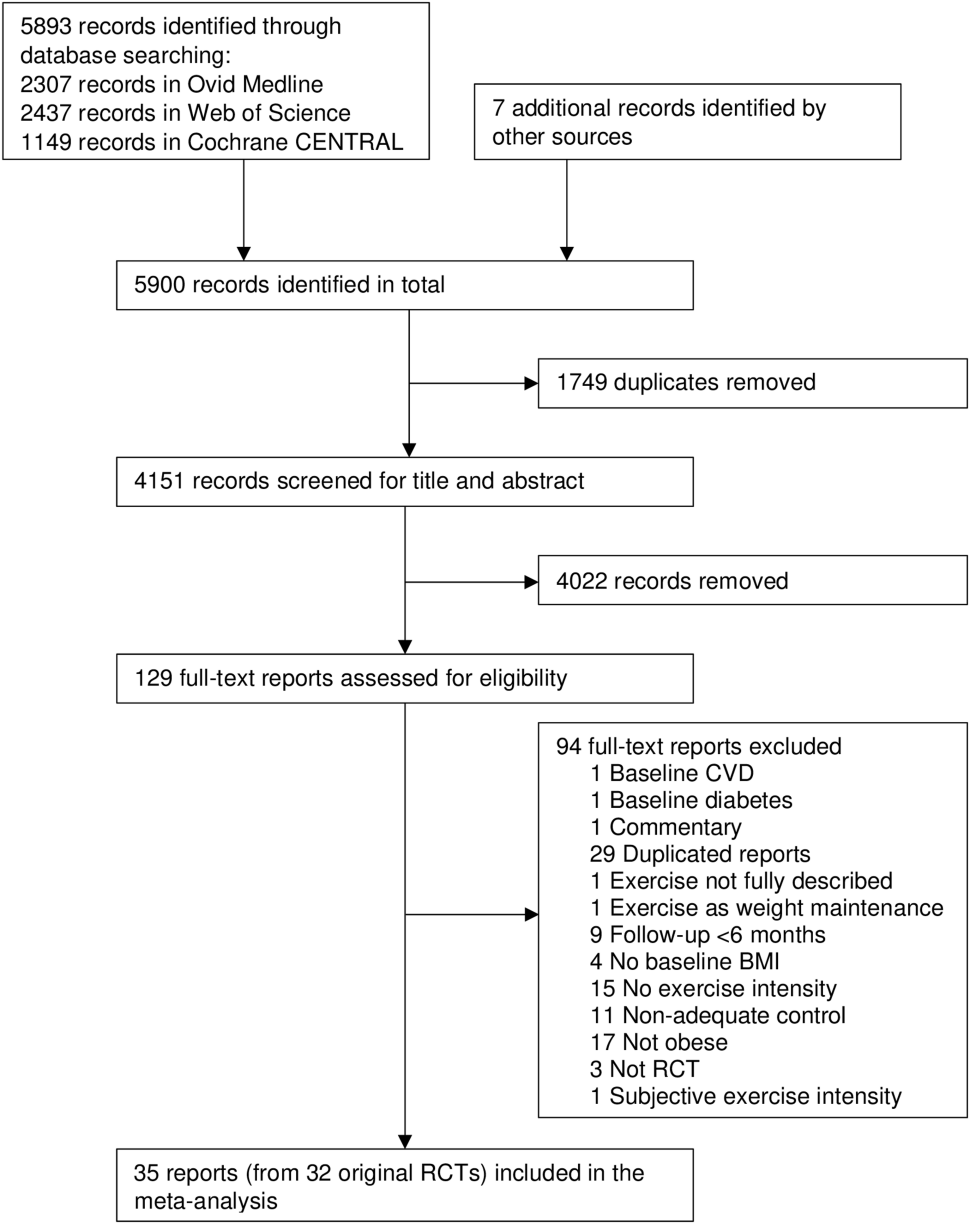
Flow diagram of the search and study selection process

Figure 1 presents the process of study search and selection. Database searches revealed 5893 records, and an additional seven were identified by checking reference lists. After the removal of duplicates, we screened 4151 reports for title and abstract. At this step, 4022 reports were excluded, leaving 129 reports assessed in full text for eligibility. We have excluded 94 full‐text reports for reasons provided in Table S4. In the end, 35 reports^16^, ^24^, ^25^, ^26^, ^27^, ^45^, ^46^, ^47^, ^48^, ^49^, ^50^, ^51^, ^52^, ^53^, ^54^, ^55^, ^56^, ^57^, ^58^, ^59^, ^60^, ^61^, ^62^, ^63^, ^64^, ^65^, ^66^, ^67^, ^68^, ^69^, ^70^, ^71^, ^72^, ^73^, ^74^ (from 32 RCTs) met our eligibility criteria and were included in the current systematic review and network meta‐analysis (excluded arms with reasons are indicated in Table S4).

### 3.1 Participants characteristics

Table S5 summarizes the characteristics of identified studies. Overall, 32 eligible RCTs included 4774 participants. All studies have a parallel design. The sample size of the studies ranged from 18 to 464. Almost two‐thirds of included studies were set in the United States,^16^, ^24^, ^27^, ^45^, ^47^, ^48^, ^51^, ^52^, ^54^, ^55^, ^56^, ^57^, ^59^, ^60^, ^62^, ^65^, ^66^, ^68^, ^69^, ^70^, ^71^, ^72^, ^73^ four in Canada,^46^, ^49^, ^53^, ^63^ two in Tunisia,^58^, ^64^ two in Korea,^50^, ^61^ and one in China,^74^ Denmark,^25^ and Israel.^26^ The participants' follow‐up lasted 6 months in 19 studies,^16^, ^25^, ^27^, ^45^, ^46^, ^47^, ^49^, ^51^, ^52^, ^53^, ^56^, ^58^, ^61^, ^62^, ^63^, ^64^, ^65^, ^67^, ^69^, ^73^ 8 months in one study,^66^, ^71^ 9 months in one study,^50^ 10 months in one study,^54^ 12 months in seven studies,^26^, ^48^, ^55^, ^57^, ^68^, ^70^, ^72^, ^74^ and 18 months in three studies.^24^, ^59^, ^60^ The mean age of participants ranged from 23.4 to 74.1 years. More than one‐third of studies were conducted in older adults (mean age ≥65 years).^16^, ^24^, ^48^, ^53^, ^56^, ^59^, ^60^, ^61^, ^65^, ^68^ Mean baseline BMI varied from 30.0 to 37.2 kg/m^2^ (without three Asian studies). Six studies had baseline BMI ≥35 kg/m^2^ considered as WHO obesity class II.^16^, ^48^, ^52^, ^56^, ^67^, ^68^, ^70^ On average, women accounted for 71.4% of trial participants. There were 13 studies conducted solely in women^45^, ^49^, ^50^, ^51^, ^52^, ^55^, ^57^, ^58^, ^64^, ^67^, ^70^, ^73^ and three in men.^54^, ^62^, ^72^ Distribution of study characteristics between available comparisons is presented in Figure S1.

### 3.2 Intervention characteristics

Regarding exercise programs, 21 studies applied AET,^16^, ^25^, ^27^, ^45^, ^46^, ^47^, ^50^, ^51^, ^52^, ^53^, ^54^, ^55^, ^58^, ^62^, ^63^, ^64^, ^66^, ^67^, ^69^, ^71^, ^72^, ^73^, ^74^ eight studies RT,^16^, ^24^, ^49^, ^50^, ^53^, ^62^, ^66^, ^70^ and 14 studies used CT.^16^, ^26^, ^48^, ^50^, ^53^, ^56^, ^57^, ^59^, ^60^, ^61^, ^64^, ^65^, ^66^, ^67^, ^68^, ^71^ None of the identified studies used HIIT. Twenty‐four studies delivered supervised facility training,^16^, ^24^, ^26^, ^27^, ^45^, ^46^, ^47^, ^48^, ^49^, ^50^, ^51^, ^52^, ^53^, ^54^, ^56^, ^61^, ^62^, ^63^, ^64^, ^65^, ^66^, ^67^, ^68^, ^69^, ^70^, ^71^, ^72^, ^73^, ^74^ four studies combined supervised facility and remote training,^55^, ^57^, ^59^, ^60^ and one study used remote training alone.^25^ Maintaining usual physical activity or prohibiting participation in formal exercise served as a control group in 28 studies.^24^, ^25^, ^26^, ^45^, ^46^, ^47^, ^48^, ^49^, ^50^, ^51^, ^52^, ^53^, ^54^, ^55^, ^56^, ^58^, ^59^, ^61^, ^62^, ^63^, ^64^, ^65^, ^66^, ^67^, ^68^, ^69^, ^70^, ^71^, ^72^, ^73^, ^74^ Minimal intervention if the form of stretching exercise or lifestyle education was used as a comparator in three studies.^27^, ^57^, ^60^

AET was delivered using aerobic machines (i.e., treadmill walking/jogging and ergometer cycling),^16^, ^27^, ^45^, ^46^, ^48^, ^50^, ^51^, ^52^, ^53^, ^54^, ^58^, ^63^, ^66^, ^68^, ^71^, ^73^ a combination of aerobic machines, and other activities (i.e., walking, jogging, and bicycling),^47^, ^55^, ^57^, ^62^, ^67^, ^69^, ^74^ jumping exercise,^64^ or walking/brisk walking/jogging.^59^, ^60^, ^61^, ^72^ Four studies did not specify what type of activity was used.^25^, ^26^, ^56^, ^65^ The duration of single aerobic sessions ranged from 20 to 60 min. Exercise intensity for AET was prescribed by % of heart rate reserve, % of maximum heart rate, % of peak heart rate, % of VO_2_ reserve, % of maximum VO_2_, or % of peak VO_2_.

All studies with RT protocols provided various exercises on major muscle groups. The number of sets ranged from one to four and the number of repetitions from six to 15. Target intensity was identified by measuring % of repetition maximum and varied from 40% to 100%.

Participants exercised with a frequency of three days per week in almost half of the studies (15 studies), 3 to 4 days per week in four studies, 4 days per week in three studies, in one study 3 to 5 days per week, and 5 days per week in seven studies. One study set training frequency to reach targeted energy expenditure.

Dietary co‐interventions with calorie restriction were adopted in 17 studies,^16^, ^24^, ^26^, ^48^, ^49^, ^50^, ^55^, ^56^, ^58^, ^59^, ^60^, ^62^, ^65^, ^67^, ^70^, ^72^, ^73^ and additionally in nine studies, those were combined with behavioral sessions or health education.^24^, ^26^, ^49^, ^50^, ^55^, ^56^, ^59^, ^60^, ^65^ Three studies used health education as a sole co‐intervention.^53^, ^57^, ^74^

**FIGURE 2:**
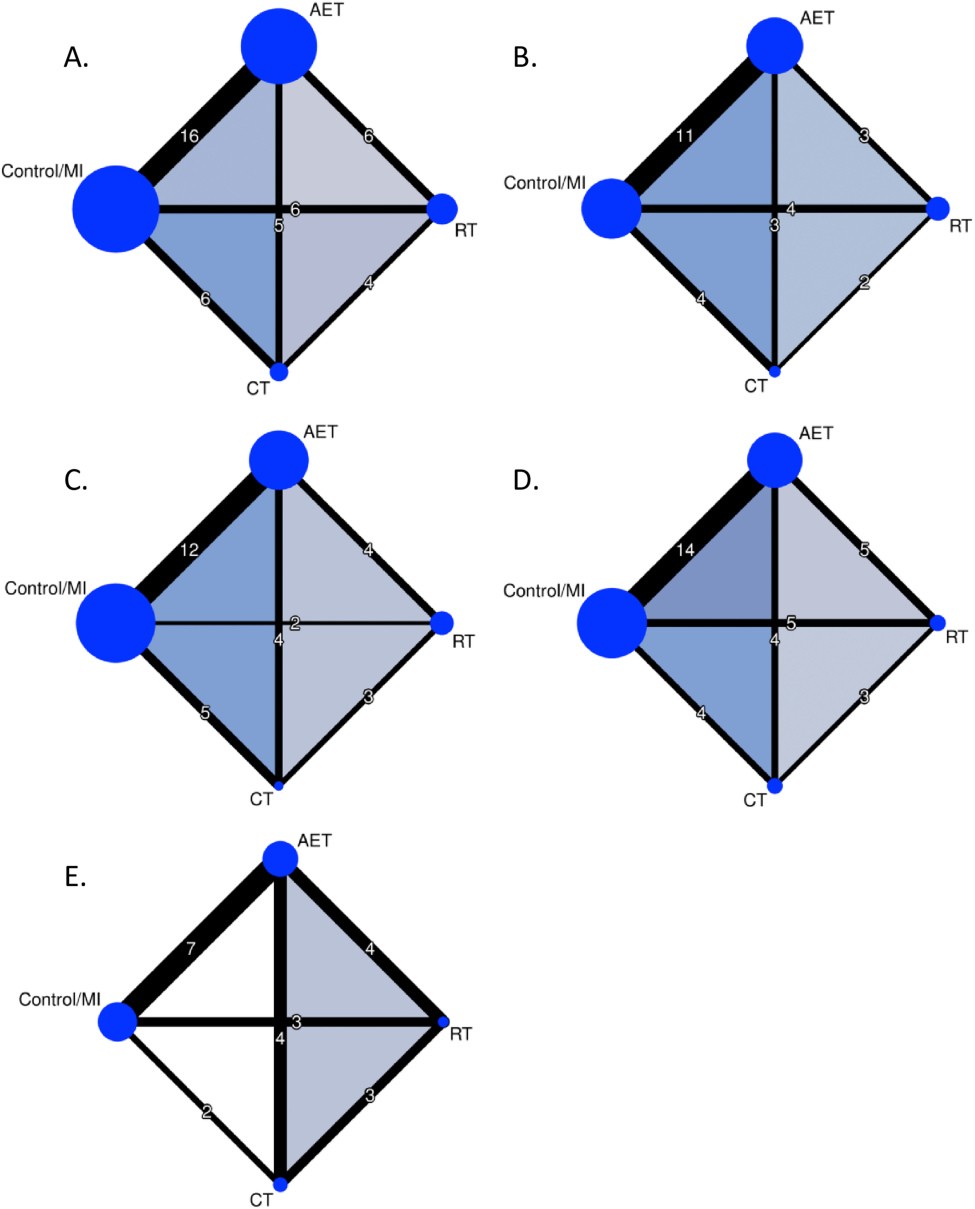
Net graphs for (A) body weight, (B) body mass index, (C) waist circumference, (D) fat mass, and (E) fat‐free mass. The size of the nodes is proportional to the total number of participants allocated to exercise modality, and the thickness of the lines is proportional to the number of studies evaluating each direct comparison. AET, aerobic exercise training; CT, combined training; MI, minimal intervention; RT, resistance training

Figure 2A–E shows available direct comparisons for BW (30 studies), BMI (20 studies), WC (21 studies), FM (24 studies), and FFM (17 studies).

### 3.3 Risk of bias

Figure S2 provides the risk of bias assessments for included RCTs. Overall, nine (26%) of the reports were rated as low, 13 (37%) as some concerns, and 13 (37%) as high risk of bias. Nineteen reports^16^, ^26^, ^27^, ^45^, ^48^, ^50^, ^51^, ^52^, ^53^, ^54^, ^57^, ^59^, ^60^, ^63^, ^65^, ^68^, ^72^ (54%) described randomization methods and allocation concealment in sufficient detail to be judged at low risk of bias arising from the randomization process. Despite that blinding of participants was not applicable, 13 reports^16^, ^26^, ^45^, ^48^, ^51^, ^53^, ^55^, ^57^, ^61^, ^63^, ^65^, ^68^, ^74^ (37%) were judged to be at low risk of bias as providing intention‐to‐treat estimates and reporting high adherence (≥80%) to exercise programs. Twenty reports^16^, ^25^, ^26^, ^27^, ^47^, ^48^, ^51^, ^53^, ^54^, ^55^, ^56^, ^57^, ^58^, ^59^, ^61^, ^63^, ^64^, ^65^, ^68^, ^74^ (57%) were judged to be at low risk of bias due to missing outcome data. Thirty‐one reports^16^, ^24^, ^25^, ^26^, ^27^, ^45^, ^46^, ^48^, ^49^, ^50^, ^51^, ^52^, ^53^, ^54^, ^55^, ^56^, ^57^, ^59^, ^60^, ^61^, ^62^, ^63^, ^64^, ^65^, ^67^, ^68^, ^69^, ^71^, ^72^, ^73^, ^74^ (89%) used appropriate anthropometric assessment methods to be judged as a low risk of bias in the measurement of outcomes. As designed and analyzed according to prespecified protocols, 20 reports^16^, ^24^, ^25^, ^26^, ^27^, ^45^, ^46^, ^48^, ^49^, ^51^, ^53^, ^54^, ^55^, ^57^, ^62^, ^63^, ^64^, ^66^, ^68^, ^74^ (57%) were judged to be at low risk of bias in the selection of reported results.

### 3.4 Body weight

**FIGURE 3:**
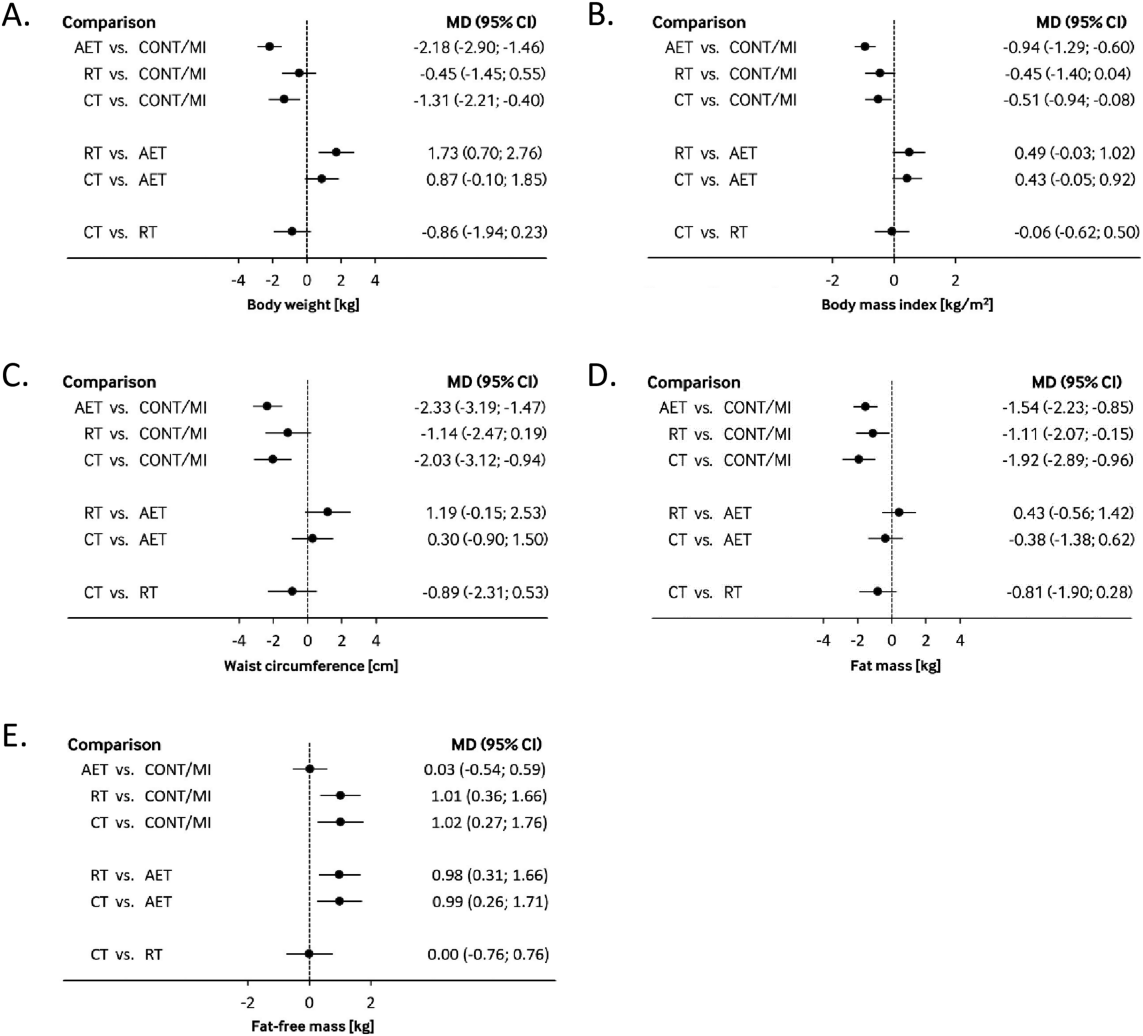
Interval plot summarizing mean difference with 95% confidence interval for (A) body weight, (B) body mass index, (C) waist circumference, (D) fat mass, and (E) fat‐free mass as estimated from the network meta‐analysis for every possible pair of training modality. Negative values favor intervention on the left side. AET, aerobic exercise training; CONT, control; CT, combined training; MI, minimal intervention; RT, resistance training

AET (MD: −2.18 kg; 95% CI: −2.90, −1.46, moderate CoE) and CT (MD: −1.31 kg; 95% CI: −2.21, −0.40, low CoE) is likely to be more effective in BW reduction than CONT/MI. We observed no benefit of CT over AET (moderate CoE). RT (MD: −0.45 kg; 95% CI: −1.45, 0.55, low CoE) was not effective for body weight loss compared to CONT/MI (Figure 3A, Table S6).

### 3.5 Body mass index

In contrast to CONT/MI, AET (MD: −0.94 kg/m^2^; 95% CI: −1.29, −0.60, low CoE), and CT (MD: −0.51 kg/m^2^; 95% CI: −0.94, −0.08, low CoE), but not RT (MD: −0.45 kg/m^2^; 95% CI: −0.94, 0.04, low CoE) show a greater decrease in BMI. (Figure 3B, Table S7).

### 3.6 Waist circumference

AET (MD: −2.33 cm; 95% CI: −3.19, −1.47, low CoE) and CT (MD: −2.03 cm; 95% CI: −3.12, −0.94, moderate CoE) are likely to improve WC when compared with CONT/MI. CT was not superior to AET in decreasing waist circumference (moderate CoE). We observed no effect when RT (MD: −1.14 cm; 95% CI: −2.47, 0.19, low CoE) was compared with CONT/MI (Figure 3C, Table S8).

### 3.7 Fat mass

We found moderate CoE that AET (MD: −1.54 kg; 95% CI: −2.23, −0.85) and CT (MD: −1.92 kg; 95% CI: −2.89, −0.96) reduces FM compared with no exercise or MI. Moreover, we observed a potential decrease in FM using RT (MD: −1.11 kg; 95% CI: −2.07, −0.15, low CoE) compared with CONT/MI (Figure 3D, Table S9).

### 3.8 Fat‐free mass

CT (MD: 1.02 kg; 95% CI: 0.27, 1.76, moderate CoE) and RT (MD: 1.01 kg; 95% CI: 0.36, 1.66, very low CoE) improved fat‐free mass compared with CONT/MI. AET (MD: 0.03 kg; 95% CI: −0.54, 0.59 kg, moderate CoE) has no effect on fat‐free mass compared with no exercise or MI (Figure 3E, Table S10). We observed that AET compared with RT (MD: −0.98 kg; 95% CI: −1.66, −0.31, low CoE) or CT (MD: −0.99 kg; 95% CI: −1.71, −0.26, moderate CoE) presents lower FFM. Moderate CoE suggests no difference in effect on FFM between CT and RT.

### 3.9 Ranking of training effectiveness

According to P‐score values, AET was ranked as the most effective for reducing body weight (0.99), BMI (0.98), and waist circumference (0.88). CT was ranked as best for fat mass (0.90). CT and RT were equally most effective for improving FFM (0.83). When we combined rankings for all outcomes (assuming their equal importance), AET was ranked as best (0.74), followed by CT (0.72), RT (0.49) and CONT/MI (0.05) (Table S11).

### 3.10 Inconsistency

The node‐splitting approach did not suggest important differences between direct and indirect estimates in any of the considered comparisons. Net heat plots suggested the presence of moderate inconsistency for all five outcomes **(**Table S12, Figures S3–S7).

### 3.11 Publication bias

Visual examination of comparison‐adjusted funnel plots (Figures S8–S12) did not suggest serious asymmetry for any of the outcomes. Similarly, results of Egger's linear regression tests provided no evidence for the presence of small‐study effects.

### 3.12 Subgroup and sensitivity analyses

Results of subgroup and sensitivity analyses are provided in Tables S13–S42. Analysis stratified for sex could not be conducted due to the low number of studies. We generally observed similar patterns in results when analyses were stratified for the length of follow‐up, initial BMI, age, and use of dietary‐cointervention. Exclusion of studies with a high risk of bias or splitting control and minimal intervention as separate nodes confirmed the main analyses' findings. Table S43 summarizes between‐study heterogeneity in subgroup and sensitivity analyses. We observed more consistent findings when focusing exclusively on participants with a higher BMI, followed for a longer period, as well as following a dietary co‐intervention.

## 4 DISCUSSION

This systematic review and NMA summarized data from 32 RCTs comparing different training modalities on anthropometric outcomes in participants with obesity. There is low to moderate certainty of evidence that both aerobic exercise and its combination with resistance exercise compared with the control or minimal intervention can reduce BW, BMI, WC, and FM. However, with moderate certainty of evidence, we consistently observed no difference in effect when combined training was compared with aerobic exercise alone. Low certainty of evidence suggested that resistance training alone reduces FM. Both resistance and combined training increased FFM by approximately 1 kg when compared with aerobic training and control or minimal intervention.

### 4.1 Comparison with other studies

This is the first NMA on the long‐term effects of different training modalities on anthropometric outcomes in obesity. Our findings for the effects of aerobic training generally correspond to previously published pairwise meta‐analyses. A meta‐analysis by *Thorogood* and colleagues including long‐term RCTs in patients with overweight and obese found that aerobic training compared with sedentary control lead to a slightly lower reduction in BW (−1.70 kg) than in our analysis (−2.18 kg), but comparable for WC (−1.95 cm vs. −2.33 cm).^12^ Likewise, in previous NMA, which pooled head‐to‐head trials comparing different training modalities, combined training was not superior over aerobic training alone for BW, BMI, and WC. However, in contrast to these findings, we could not observe differences between aerobic (except for BW) and combined training when compared with resistance training.^21^ Results from that NMA can be explained by short follow‐up, additional inclusion of overweight patients, and missing evidence from studies with sedentary control. With respect to body fat, we observed slightly greater decrease in combined (−1.92 kg) than in aerobic (−1.54 kg) and resistance (−1.11 kg) training. This supports findings from a recent meta‐analysis suggesting the superiority of combined over aerobic and resistance training alone in the reduction of subcutaneous adipose tissue, which is the biggest compartment of body fat.^13^

The effects of aerobic exercise on anthropometric outcomes observed in the current review can be explained by an increase in activity energy expenditure, leading to negative energy balance.^75^ Consistently, resistance training is suggested to introduce smaller energy expenditure changes, leading to less effects on anthropometric outcomes.^76^ However, resistance exercises induce post‐exercise oxygen consumption and fat beta‐oxidation,^77^, ^78^ which might contribute to the observed further decrease in FM beyond an increase in overall energy expenditure. Promotion of FFM increase with resistance training might depend on exercise intensity and volume.^79^ Despite this, we observed a beneficial effect of resistance and combined training on improving FFM.

A crucial factor influencing the exercise effect on weight loss is the reduction of caloric intake. However, our results indicated that effects of aerobic exercise on BW (−2.29 kg vs. −1.80 kg), WC (−2.36 cm vs. −2.21 cm), and FM (−1.32 kg vs. −1.63 kg) were comparable irrespective of dietary co‐intervention use. A similar remark was made in a narrative review by *Chin* and colleagues suggesting that aerobic training alone allows achieving recommended weight loss goals, but this is likely dose‐dependent on exercise intensity.^76^

### 4.2 Clinical and research implications

Aerobic training programs included in the present systematic review and NMA met the duration, frequency, and intensity goals suggested by available recommendations. Long‐term adherence to regular moderate‐to‐vigorous aerobic training produced a meaningful reduction in BW, BMI, WC, and FM compared with non‐exercising. Therefore, our results are supportive for current weight management guidelines.^6^, ^7^, ^8^, ^9^, ^10^, ^11^ Even though aerobic training's beneficial effects were found to be irrespective of co‐administration of dietary intervention, exercising should be combined with caloric restriction to increase the magnitude of weight loss. When applied alone, resistance training seemed to be effective for selected anthropometry outcomes but with unsure marginal effectiveness for long‐term weight loss in contrast to aerobic training, and thus, it should not be recommended unequivocally. Combining aerobic and resistance training might induce a greater reduction of FM. Additional improvement in FFM as a consequence of resistance and combined exercises is highly desired, especially in older patients. Moreover, adding resistance training to aerobic training might provide additional benefits in respect to blood pressure, cardiorespiratory fitness, glycemic control, or lipid profile, and thus, it should be considered in the context of co‐morbidities present in obesity.^80^

When transferring the current results, we should consider that identified RCTs included patients with moderate obesity predominately. Therefore, we had a limited ability to conclude on effects of training interventions in overweight or severe obesity. Moreover, as most included exercise protocols focused on supervised, facility‐based sessions, it is hard to refer these findings to the benefits of home‐based programs or leisure physical activity.

Our systematic review did not identify any long‐term RCTs comparing the effects of HIIT on anthropometric outcomes in obesity. Thus, considering promising findings suggested by evidence from short‐term studies,^18^, ^19^ there is an urgent need for long‐term trials, which will allow advocating whether HIIT might be an effective strategy for weight loss in patients with obesity.

### 4.3 Strengths and limitations

The following strengths of the current review can be indicated: availability of predefined systematic review protocol, a comprehensive systematic search of current evidence, risk of bias assessment using the novel Cochrane RoB 2 tool, use of NMA as well as subgroup/sensitivity analyses, and application of the GRADE approach to assess the certainty of evidence.

There are several limitations that we have to consider when interpreting the results. The major one is a statistical inconsistency, which was detected for all considered outcomes. Despite potential inconsistency caused by age, baseline BMI, length of follow‐up or additional use of diet which we explored subgroup analyses, an unexplored source of inconsistency might be differences in exercise protocols. Training protocols varied in duration, frequency, and intensity of exercise. The effectiveness of weight loss is dose‐related to the volume of exercise. Some trials prescribed individualized exercise goals or changed the volume of exercise throughout the study period. Moreover, the diversity of exercise protocols description did not allow us to produce a common comparative measure that we could use in the analysis. Another limitation is the fact that our review focused only on exercise for active weight loss. Therefore, we cannot conclude on longitudinal weight maintenance or risk of regaining weight, which differs depending on the method used for weight loss.^81^, ^82^ More than one‐third of trials were at high risk of bias due to deviation from intended interventions or missing outcomes, but a sensitivity analysis revealed that exclusion of them did not have a large impact. The selective choice of reported outcome measures was likely to influence our analysis. For example, BMI is highly correlated with body weight, but it was less frequently available in studies. This could explain situations when findings for body weight did not match with those for BMI.

## 5 CONCLUSIONS

We identified low to moderate certainty of evidence that aerobic training improves body weight, BMI, waist circumference, and fat mass compared with control or minimal intervention. In this setting, resistance training reduced fat mass and improved fat‐free mass. Comparable effects of combined training relative to aerobic training with respect to body weight, BMI, and waist circumference might be explained by aerobic, but not resistance component. However, the addition of resistance training to aerobic exercise has an additional individual beneficial impact on fat mass reduction and on the fat‐free mass increase.

## FUNDING INFORMATION

The research work of A.D. and K.P. was financially supported by the Ministry of Science and Higher Education (Ministerstwo Nauki i Szkolnictwa Wyższego) in the range of the program entitled “Regional Initiative of Excellence” for the years 2019 to 2022, project no. 010/RID/2018/19, amount of funding 12,000,000 PLN.

## CONFLICT OF INTEREST

J.M, G.R., A.D., K.P., M.N., S.S., and L.S. declare that they have no conflict of interest.

## AUTHORS' CONTRIBUTIONS

J.M., K.P., and L.S. conceptualized the idea for the present review, and J.M., A.D., M.N., S.S., and L.S. collected the data (including the risk of bias assessment). J.M. and G.R. analyzed the data. J.M. and L.S. wrote the first draft. All authors reviewed and commented on subsequent drafts of the manuscript.

## Supporting information


**Table S1.** Example of strategy used for the search of MEDLINE® (OvidSP).
**Table S2.** Additional data received by e‐mail contact with primary/corresponding authors of included studies.
**Table S3.** Reasons for risk of bias assessment judgement in the present systematic review.
**Table S4.** Reasons for study and arms exclusion at the full‐text eligibility assessment.
**Table S5.** Characteristics of participants and training protocols in 32 randomized controlled trials included in the current systematic review.
**Figure S1.** Boxplots presenting the distribution of (**A**) age, (**B**) body mass index (BMI), (**C**) % of female, and (**D**) follow‐up length for different pairs of comparisons.
**Figure S2.** Summary of the risk of bias assessment for studies included in the current review.
**Table S6.** GRADE evaluation for body weight (kg) and all comparisons.*
**Table S7.** GRADE evaluation for body mass index (kg/m^2^) and all comparisons.*
**Table S8.** GRADE evaluation for waist circumference (cm) and all comparisons.*
**Table S9.** GRADE evaluation for fat mass (kg) and all comparisons.*
**Table S10.** GRADE evaluation for fat‐free mass (kg) and all comparisons.*
**Table S11.** Relative ranking* of training effects on anthropometric outcomes.
**Table S12.** Results of node‐splitting approach to assess inconsistency for anthropometric outcomes.
**Figure S3.** Net‐heat plot* to assess inconsistency for body weight.
**Figure S4.** Net‐heat plot to assess inconsistency for body mass index.
**Figure S5.** Net‐heat plot to assess inconsistency for waist circumference.
**Figure S6.** Net‐heat plot to assess inconsistency for fat mass.
**Figure S7.** Net‐heat plot to assess inconsistency for fat‐free mass.
**Figure S8.** Comparison‐adjusted funnel plot for body weight.
**Figure S9.** Comparison‐adjusted funnel plot for body weight.
**Figure S10.** Comparison‐adjusted funnel plot for waist circumference.
**Figure S11.** Comparison‐adjusted funnel plot for fat mass.
**Figure S12**. Comparison‐adjusted funnel plot for fat‐free mass.
**Table S13**. Subgroup analysis presenting mean differences* with 95% CI in body weight between different training modalities, stratified by mean age: <65 years (bottom‐left) and ≥65 years (upper‐right).
**Table S14**. Subgroup analysis presenting mean differences with 95% CI in body mass index between different training modalities, stratified by mean age: <65 years (bottom‐left) and ≥65 years (upper‐right).
**Table S15**. Subgroup analysis presenting mean differences with 95% CI in waist circumference between different training modalities, stratified by mean age: <65 years (bottom‐left) and ≥65 years (upper‐right).
**Table S16**. Subgroup analysis presenting mean differences with 95% CI in fat mass between different training modalities, stratified by mean age: <65 years (bottom‐left) and ≥65 years (upper‐right).
**Table S17**. Subgroup analysis presenting mean differences with 95% CI in fat‐free mass between different training modalities, stratified by mean age: <65 years (bottom‐left) and ≥65 years (upper‐right).
**Table S18**. Subgroup analysis presenting mean differences with 95% CI in body weight between different training modalities, stratified by mean BMI: <35 kg/m^2^ (bottom‐left) and ≥35 kg/m^2^ (upper‐right).
**Table S19**. Subgroup analysis presenting mean differences with 95% CI in body mass index between different training modalities, stratified by mean BMI: <35 kg/m^2^ (bottom‐left) and ≥35 kg/m^2^ (upper‐right).
**Table S20**. Subgroup analysis presenting mean differences with 95% CI in waist circumference between different training modalities, stratified by mean BMI: <35 kg/m^2^ (bottom‐left) and ≥35 kg/m^2^ (upper‐right).
**Table S21.** Subgroup analysis presenting mean differences with 95% CI in fat mass between different training modalities, stratified by mean BMI: <35 kg/m2 (bottom‐left) and ≥35 kg/m2 (upper‐right).
**Table S22**. Subgroup analysis presenting mean differences with 95% CI in fat‐free mass between different training modalities, stratified by mean BMI: <35 kg/m^2^ (bottom‐left) and ≥35 kg/m^2^ (upper‐right).
**Table S23**. Subgroup analysis presenting mean differences with 95% CI in body weight between different training modalities, stratified by length of follow‐up: <12 months (bottom‐left) and ≥12 months (upper‐right).
**Table S24**. Subgroup analysis presenting mean differences with 95% CI in body mass index between different training modalities, stratified by length of follow‐up: <12 months (bottom‐left) and ≥12 months (upper‐right).
**Table S25**. Subgroup analysis presenting mean differences with 95% CI in waist circumference between different training modalities, stratified by length of follow‐up: <12 months (bottom‐left) and ≥12 months (upper‐right).
**Table S26**. Subgroup analysis presenting mean differences with 95% CI in fat mass between different training modalities, stratified by length of follow‐up: <12 months (bottom‐left) and ≥12 months (upper‐right).
**Table S27**. Subgroup analysis presenting mean differences with 95% CI in fat‐free mass between different training modalities, stratified by length of follow‐up: <12 months (bottom‐left) and ≥12 months (upper‐right).
**Table S28**. Subgroup analysis presenting mean differences with 95% CI in body weight between different training modalities, stratified by use of dietary co‐intervention: absent (bottom‐left) and present (upper‐right).
**Table S29**. Subgroup analysis presenting mean differences with 95% CI in body mass index between different training modalities, stratified by use of dietary co‐intervention: absent (bottom‐left) and present (upper‐right).
**Table S30**. Subgroup analysis presenting mean differences with 95% CI in waist circumference between different training modalities, stratified by use of dietary co‐intervention: absent (bottom‐left) and present (upper‐right).
**Table S31**. Subgroup analysis presenting mean differences with 95% CI in fat mass between different training modalities, stratified by use of dietary co‐intervention: absent (bottom‐left) and present (upper‐right).
**Table S32**. Subgroup analysis presenting mean differences with 95% CI in fat‐free mass between different training modalities, stratified by use of dietary co‐intervention: absent (bottom‐left) and present (upper‐right).
**Table S33**. Sensitivity analysis presenting mean differences with 95% CI in body weight between different training modalities, after exclusion of studies with overall high risk of bias.
**Table S34**. Sensitivity analysis presenting mean differences with 95% CI in body mass index between different training modalities, after exclusion of studies with overall high risk of bias.
**Table S35**. Sensitivity analysis presenting mean differences with 95% CI in weight circumference between different training modalities, after exclusion of studies with overall high risk of bias.
**Table S36**. Sensitivity analysis presenting mean differences with 95% CI in fat mass between different training modalities, after exclusion of studies with overall high risk of bias.
**Table S37**. Sensitivity analysis presenting mean differences with 95% CI in fat‐free mass between different training modalities, after exclusion of studies with overall high risk of bias.
**Table S38**. Sensitivity analysis presenting mean differences with 95% CI in body weight between different training modalities, after splitting control and minimal intervention into separate nodes.
**Table S39**. Sensitivity analysis presenting mean differences with 95% CI in body mass index between different training modalities, after splitting control and minimal intervention into separate nodes.
**Table S40**. Sensitivity analysis presenting mean differences with 95% CI in weight circumference between different training modalities, after splitting control and minimal intervention into separate nodes.
**Table S41**. Sensitivity analysis presenting mean differences with 95% CI in fat mass between different training modalities, after splitting control and minimal intervention into separate nodes.
**Table S42**. Sensitivity analysis presenting mean differences with 95% CI in fat‐free mass between different training modalities, after splitting control and minimal intervention into separate nodes.
**Table S43**. Between‐study heterogeneity (network τ^2^ and I^2^ statistic) in subgroup and sensitivity analyses.
