# Supplementary material for: Impact of different training modalities on anthropometric outcomes in patients with obesity: A systematic review and network meta‐analysis
Source: Obes Rev. 2021 Feb 23;22(7):e13218. doi: 10.1111/obr.13218 (PMC8244024; doi:10.1111/obr.13218)
Supplement: Supplementary file 1 — Table S1. Example of strategy used for the search of MEDLINE® (OvidSP). Table S2. Additional data received by e‐mail contact with primary/corresponding authors of included studies. Table S3. Reasons for risk of bias assessment judgement in the present systematic review. Table S4. Reasons for study and arms exclusion at the full‐text eligibility assessment. Table S5. Characteristics of participants and training protocols in 32 randomized controlled trials included in the current systematic review. Figure S1. Boxplots presenting the distribution of (A) age, (B) body mass index (BMI), (C) % of female, and (D) follow‐up length for different pairs of comparisons. Figure S2. Summary of the risk of bias assessment for studies included in the current review. Table S6. GRADE evaluation for body weight (kg) and all comparisons.* Table S7. GRADE evaluation for body mass index (kg/m2) and all comparisons.* Table S8. GRADE evaluation for waist circumference (cm) and all comparisons.* Table S9. GRADE evaluation for fat mass (kg) and all comparisons.* Table S10. GRADE evaluation for fat‐free mass (kg) and all comparisons.* Table S11. Relative ranking* of training effects on anthropometric outcomes. Table S12. Results of node‐splitting approach to assess inconsistency for anthropometric outcomes. Figure S3. Net‐heat plot* to assess inconsistency for body weight. Figure S4. Net‐heat plot to assess inconsistency for body mass index. Figure S5. Net‐heat plot to assess inconsistency for waist circumference. Figure S6. Net‐heat plot to assess inconsistency for fat mass. Figure S7. Net‐heat plot to assess inconsistency for fat‐free mass. Figure S8. Comparison‐adjusted funnel plot for body weight. Figure S9. Comparison‐adjusted funnel plot for body weight. Figure S10. Comparison‐adjusted funnel plot for waist circumference. Figure S11. Comparison‐adjusted funnel plot for fat mass. Figure S12. Comparison‐adjusted funnel plot for fat‐free mass. Table S13. Subgroup analysis presenting mean d [file OBR-22-e13218-s001.pdf]

Impact of different training modalities on anthropometric outcomes in patients with obesity: a systematic review and network meta-analysis

Jakub Morze[1,2], Gerta Rücker[3], Anna Danielewicz[2], Katarzyna Przybyłowicz[2], Manuela Neuenschwander[4], Sabrina Schlesinger[4], Lukas Schwingshackl[5]

[1] Department of Cardiology and Internal Diseases, University of Warmia and Mazury, Olsztyn, Poland.

[2] Department of Human Nutrition, University of Warmia and Mazury, Olsztyn, Poland.

[3] Institute of Medical Biometry and Statistics, Medical Center - University of Freiburg, Faculty of Medicine, University of Freiburg, Freiburg, Germany.

[4] Institute for Biometrics and Epidemiology, German Diabetes Center (DDZ), Leibniz Center for Diabetes Research at Heinrich Heine University Düsseldorf, Düsseldorf, Germany.

[5] Institute for Evidence in Medicine, Medical Center - University of Freiburg, Faculty of Medicine, University of Freiburg, Freiburg, Germany

Corresponding author: Jakub Morze

al. Warszawska 30, 10-082, Olsztyn, Poland

Phone: +48 517 571 494

E-Mail: jakub.morze@uwm.edu.pl

ONLINE SUPPORTING INFORMATION

Online Supporting Information

Content	Pages
Table S1. Search strategy.	3
Table S2. Additional data received from authors.	4
Table S3. Reasons for risk of bias assessment judgement.	5
Table S4. Reasons for study full-text and study arms exclusion and supplementary references.	6-9
Table S5. Study characteristics.	11-17
Figure S1. Box plots.	18
Figure S2. Risk of bias.	19-20
Table S6-10. GRADE evaluation.	21-25
Table S11. Relative ranking.	26
Table S12. Node-splitting approach.	26
Figure S3-7. Net-heat plots.	27-31
Figure S8-12. Comparison-adjusted funnel plots.	32-34
Table S13-32. Subgroup analysis for network meta-analysis model.	35-38
Table S33-42. Sensitivity analysis for network meta-analysis model.	39-40
Table S43. Between-study heterogeneity in subgroup/sensitivity analyses.	41

Online Supporting Information

Table S1. Example of strategy used for the search of MEDLINE® (OvidSP).

1	Diet, Reducing/
2	(weight reduction OR weight loss OR weight maintanance).ti,ab.
3	Weight Loss/
4	#1 OR #2 OR #3
5	Obesity/th [Therapy]
6	Overweight/
7	(obese OR obesity OR overweight).ti,ab.
8	#5 OR #6 OR #7
9	exp "Physical Education and Training"/
10	exercis*.tw.
11	exp Exercise/
12	(physic* adj1 (activ* OR fit*)).ti,ab.
13	(((physic* OR strength* OR resist* OR circuit OR weight OR aerob* OR cross OR endurance OR structur* OR combined OR interval) adj3 train*) OR high-intens* OR hight intens*).ti,ab.
14	#9 OR #10 OR #11 OR #12 OR #13
15	randomi?ed.ab.
16	placebo.ab.
17	drug therapy.fs.
18	randomly.ab.
19	trial.ab.
20	groups.ab.
21	15 OR 16 OR 17 OR 18 OR 19 OR 20
22	exp animals/ NOT humans/
23	#21 NOT #22
24	#4 AND #8 AND #14 AND #23
25	24 and (adults OR men OR women OR man OR woman OR adulthood OR adult).ti,ab.

Table S2. Additional data received by e-mail contact with primary/corresponding authors of included studies.

Reference	Data received
Donnelly et al. 2013	Waist circumference (change from baseline in treatment arms)
Gram et al. 2017	Fat mass, fat-free mass, waist circumference (pre- and post-intervention means in treatment arms)
Gepner et al.2017	Body weight, body mass index, weight circumference (pre- and post-intervention means in treatment arms)
Villareal et al.2011	Body mass index (change from baseline in treatment arms)
Villareal et al.2017	Body mass index (change from baseline in treatment arms)
Watkins et al. 2003	Waist circumference (pre- and post-intervention means in treatment arms)

Online Supporting Information

Table S3. Reasons for risk of bias assessment judgement in the present systematic review.

Cochrane RoB2 domain	Domain-level judgements	Reasons for judgement
Bias arising from the randomization process	Low risk	Available description of random sequence generation and allocation concealment (in the manuscript or trial protocol) as well as no evidence for baseline imbalances.
	Some concerns	Missing description of allocation concealment, but no evidence for baseline imbalances.
	High risk	Missing description of allocation concealment but there is an evidence that baseline imbalances were due to failure to proper random allocation of participants.
Bias due to deviations from the intended interventions	Low risk	Study used ITT (or mITT) approach and reported high adherence of participants to exercise programs (i.e ≥80% of session completed).
	Some concerns	Study used ITT (mITT) or per-protocol approach and reported moderate non-adherence of participants to exercise program (i.e. <80% of sessions completed).
	High risk	Study used per-protocol approach in case of serious non-adherence or unwillingness to continue exercise program.
Bias due to missing outcome data	Low risk	Study's drop-out rate ≤20%, with stated reasons, and dropouts were likely not dependent on their true value.
	Some concerns	Study's drop-out rate >20%, but reasons for dropouts missing, and dropouts were likely not dependent on their true value.
	High risk	Study's drop-out rate >20%, and dropouts were likely dependent on their true value (i.e., rates not equal between groups).
Bias in the measurement of the outcome	Low risk	Anthropometrics and body composition assessed using recommended reliable methodology (i.e. DXA, MRI)
	Some concerns	Anthropometrics and body composition assessed using less reliable methodology (i.e. bioimpedance analysis)
	High risk	NA
Bias in the selection of the reported result	Low risk	No evidence for results selection and pre-specified trial protocol available.
	Some concerns	No evidence for results selection but missing pre-specified trial protocol available.
	High risk	Presence of evidence for results selection.

Abbreviations: DXA – dual energy x-ray absorptiometry, ITT – intention-to-treat, mITT – modified intention-to-treat, MRI – magnetic resonance imaging; NA – not applicable;
Presented judgements are based on assessment algorithms available in the guidance document for the revised Cochrane risk-of-bias tool for randomized trials (RoB2).

Online Supporting Information

Table S4. Reasons for study and arms exclusion at the full-text eligibility assessment.

Reference	Reasons for exclusion of studies
1	Baseline cardiovascular disease
2	Baseline type 2 diabetes
3	Commentary
4-32	Duplicated report
33-47	Exercise intensity not provided
48	Exercise protocol not fully described
49	Exercise used as a weight maintaining protocol
50-58	Follow-up lasting less than 6 months
59-62	No information on baseline BMI
63-73	Non-adequate control intervention
74-90	Not obese (mean baseline BMI<30 kg/m^2 or <25 kg/m^2 in Asian studies)
91-93	Not randomised/quasi-randomised trial
94	Subjective intensity of exercise (Borg Scale)
**Reference**	**Reasons for exclusion of arms from included studies**
**95-99**	Subjective intensity of exercise
**100-103**	Unbalanced co-intervention
**99 104 105**	Not obese (arm-level mean baseline BMI <30 kg/m^2 or <25 kg/m^2 in Asian studies)

References:

1. Beavers KM, Ambrosius WT, Nicklas BJ, et al. Independent and combined effects of physical activity and weight loss on inflammatory biomarkers in overweight and obese older adults. *Journal of the American Geriatrics Society* 2013;61(7):1089-94.
2. Daly RM, Dunstan DW, Owen N, et al. Does high-intensity resistance training maintain bone mass during moderate weight loss in older overweight adults with type 2 diabetes? *Osteoporosis International* 2005;16(12):1703-12.
3. Chambliss HO. Exercise duration and intensity in a weight-loss program. *Clinical Journal of Sport Medicine* 2005;15(2):113-5.
4. Abbenhardt C, McTiernan A, Alfano CM, et al. Effects of individual and combined dietary weight loss and exercise interventions in postmenopausal women on adiponectin and leptin levels. *Journal of Internal Medicine* 2013;274(2):163-75.
5. Armamento-Villareal R, Aguirre L, Napoli N, et al. Changes in thigh muscle volume predict bone mineral density response to lifestyle therapy in frail, obese older adults. *Osteoporosis International* 2014;25(2):551-8.
6. Armamento-Villareal R, Aguirre LE, Qualls C, et al. Effect of Lifestyle Intervention on the Hormonal Profile of Frail, Obese Older Men. *Journal of Nutrition, Health & Aging* 2016;20(3):334-40.
7. Armamento-Villareal R, Sadler C, Napoli N, et al. Weight loss in obese older adults increases serum sclerostin and impairs hip geometry but both are prevented by exercise training. *Journal of Bone & Mineral Research* 2012;27(5):1215-21.
8. Bales CW, Hawk VH, Granville EO, et al. Aerobic and Resistance Training Effects on Energy Intake: The STRRIDE-AT/RT Study. *Medicine and Science in Sports and Exercise* 2012;44(10):2033-39. doi: 10.1249/MSS.0b013e318259479a
9. Beavers DP, Beavers KM, Loeser RF, et al. The independent and combined effects of intensive weight loss and exercise training on bone mineral density in overweight and obese older adults with osteoarthritis. *Osteoarthritis & Cartilage* 2014;22(6):726-33.
10. Beavers KM, Beavers DP, Newman JJ, et al. Effects of total and regional fat loss on plasma CRP and IL-6 in overweight and obese, older adults with knee osteoarthritis. *Osteoarthritis & Cartilage* 2015;23(2):249-56.
11. Beavers KM, Walkup MP, Weaver AA, et al. Effect of Exercise Modality During Weight Loss on Bone Health in Older Adults With Obesity and Cardiovascular Disease or Metabolic Syndrome: A Randomized Controlled Trial. *Journal of Bone & Mineral Research* 2018;33(12):2140-49.
12. Campbell DD, Meckling KA. Effect of the protein:carbohydrate ratio in hypoenergetic diets on metabolic syndrome risk factors in exercising overweight and obese women. *British Journal of Nutrition* 2012;108(9):1658-71.
13. Chua SD, Jr., Messier SP, Legault C, et al. Effect of an exercise and dietary intervention on serum biomarkers in overweight and obese adults with osteoarthritis of the knee. *Osteoarthritis & Cartilage* 2008;16(9):1047-53.
14. Chubak J, Ulrich CM, Tworoger SS, et al. Effect of exercise on bone mineral density and lean mass in postmenopausal women. *Medicine and Science in Sports and Exercise* 2006;38(7):1236-44. doi: 10.1249/01.mss.0000227308.11278.d7
15. Church TS, Martin CK, Thompson AM, et al. Changes in weight, waist circumference and compensatory responses with different doses of exercise among sedentary, overweight postmenopausal women. *PLoS ONE [Electronic Resource]* 2009;4(2):e4515.

16. Drapeau S, Doucet E, Rabasa-Lhoret R, et al. Improvement in insulin sensitivity by weight loss does not affect hyperinsulinemia-mediated reduction in total and high molecular weight adiponectin: a MONET study. *Applied Physiology, Nutrition, & Metabolism = Physiologie Appliquee, Nutrition et Metabolisme* 2011;36(2):191-200.
17. Georgiades A, Sherwood A, Gullette EC, et al. Effects of exercise and weight loss on mental stress-induced cardiovascular responses in individuals with high blood pressure. *Hypertension* 2000;36(2):171-6.
18. Hammond BP, Stotz PJ, Brennan AM, et al. Individual Variability in Waist Circumference and Body Weight in Response to Exercise. *Medicine & Science in Sports & Exercise* 2019;51(2):315-22.
19. Hinderliter A, Sherwood A, Gullette EC, et al. Reduction of left ventricular hypertrophy after exercise and weight loss in overweight patients with mild hypertension. *Archives of internal medicine* 2002;162(12):1333-9.
20. Hintze LJ, Messier V, Lavoie ME, et al. A one-year resistance training program following weight loss has no significant impact on body composition and energy expenditure in postmenopausal women living with overweight and obesity. *Physiology & Behavior* 2018;189:99-106.
21. Hochsmann C, Dorling JL, Apolzan JW, et al. Effect of different doses of supervised aerobic exercise on heart rate recovery in inactive adults who are overweight or obese: results from E-MECHANIC. *European journal of applied physiology* 2019;119(9):2095-103.
22. Imayama I, Ulrich CM, Alfano CM, et al. Effects of a caloric restriction weight loss diet and exercise on inflammatory biomarkers in overweight/obese postmenopausal women: a randomized controlled trial. *Cancer Research* 2012;72(9):2314-26.
23. Kiernan M, King AC, Stefanick ML, et al. Men gain additional psychological benefits by adding exercise to a weight-loss program. *Obesity Research* 2001;9(12):770-7.
24. Messier SP, Loeser RF, Miller GD, et al. Exercise and dietary weight loss in overweight and obese older adults with knee osteoarthritis: the Arthritis, Diet, and Activity Promotion Trial. *Arthritis & Rheumatism* 2004;50(5):1501-10.
25. Messier V, Rabasa-Lhoret R, Doucet E, et al. Effects of the addition of a resistance training programme to a caloric restriction weight loss intervention on psychosocial factors in overweight and obese post-menopausal women: a Montreal Ottawa New Emerging Team study. *Journal of Sports Sciences* 2010;28(1):83-92.
26. Miller GD, Beavers DP, Hamm D, et al. Nutrient Intake During Diet-Induced Weight Loss and Exercise Interventions in a Randomized Trial in Older Overweight and Obese Adults. *Journal of Nutrition, Health & Aging* 2017;21(10):1216-24.
27. Rejeski WJ, Ambrosius WT, Burdette JH, et al. Community Weight Loss to Combat Obesity and Disability in At-Risk Older Adults. *Journals of Gerontology Series A-Biological Sciences & Medical Sciences* 2017;72(11):1547-53.
28. Rejeski WJ, Marsh AP, Fanning J, et al. Dietary Weight Loss, Exercise, and Inflammation in Older Adults with Overweight or Obesity and Cardiometabolic Disease. *Obesity* 2019;27(11):1805-11.
29. Stewart LK, Earnest CP, Blair SN, et al. Effects of different doses of physical activity on C-reactive protein among women. *Medicine & Science in Sports & Exercise* 2010;42(4):701-7.
30. St-Onge M, Rabasa-Lhoret R, Strychar I, et al. Impact of energy restriction with or without resistance training on energy metabolism in overweight and obese postmenopausal women: a Montreal Ottawa New Emerging Team group study. *Menopause* 2013;20(2):194-201.
31. Swift DL, Johannsen NM, Earnest CP, et al. Effect of Different Doses of Aerobic Exercise Training on Total Bilirubin Levels. *Medicine and Science in Sports and Exercise* 2012;44(4):569-74. doi: 10.1249/MSS.0b013e3182357dd4
32. Taylor TR, Dash C, Sheppard V, et al. The effect of a randomized controlled physical activity trial on health related quality of life in metabolically unhealthy African-American women: FIERCE STUDY. *Contemporary Clinical Trials* 2018;67:121-28.
33. Ard JD, Carson TL, Shikany JM, et al. Weight loss and improved metabolic outcomes amongst rural African American women in the Deep South: six-month outcomes from a community-based randomized trial. *Journal of Internal Medicine* 2017;282(1):102-13.
34. Goodpaster BH, Delany JP, Otto AD, et al. Effects of diet and physical activity interventions on weight loss and cardiometabolic risk factors in severely obese adults: a randomized trial. *Jama* 2010;304(16):1795-802.
35. Gwinup G. Weight loss without dietary restriction: efficacy of different forms of aerobic exercise. *American Journal of Sports Medicine* 1987;15(3):275-9.
36. Jeffery RW, Wing RR, Sherwood NE, et al. Physical activity and weight loss: does prescribing higher physical activity goals improve outcome? *American Journal of Clinical Nutrition* 2003;78(4):684-9.
37. Jeffery RW, Wing RR, Thorson C, et al. Use of personal trainers and financial incentives to increase exercise in a behavioral weight-loss program. *Journal of Consulting & Clinical Psychology* 1998;66(5):777-83.
38. Jenkinson CM, Doherty M, Avery AJ, et al. Effects of dietary intervention and quadriceps strengthening exercises on pain and function in overweight people with knee pain: randomised controlled trial. *BMJ* 2009;339:b3170.
39. Madjd A, Taylor MA, Shafiei Neek L, et al. Effect of weekly physical activity frequency on weight loss in healthy overweight and obese women attending a weight loss program: a randomized controlled trial. *American Journal of Clinical Nutrition* 2016;104(5):1202-08.
40. Mora-Rodriguez R, Fernandez-Elias VE, Morales-Palomo F, et al. Aerobic interval training reduces vascular resistances during submaximal exercise in obese metabolic syndrome individuals. *European journal of applied physiology* 2017;117(10):2065-73. doi: 10.1007/s00421-017-3697-7
41. Nam S, Stewart KJ, Dobrosielski DA. Lifestyle Intervention for Sleep Disturbances Among Overweight or Obese Individuals. *Behavioral Sleep Medicine* 2016;14(3):343-50.

42. Skender ML, Goodrick GK, Del Junco DJ, et al. Comparison of 2-year weight loss trends in behavioral treatments of obesity: diet, exercise, and combination interventions. *Journal of the American Dietetic Association* 1996;96(4):342-6.
43. Toji C, Okamoto N, Kobayashi T, et al. Effectiveness of diet versus exercise intervention on weight reduction in local Japanese residents. *Environmental Health & Preventive Medicine* 2012;17(4):332-40.
44. Volpe SL, Kobusingye H, Bailur S, et al. Effect of diet and exercise on body composition, energy intake and leptin levels in overweight women and men. *Journal of the American College of Nutrition* 2008;27(2):195-208.
45. Wadden TA, Vogt RA, Andersen RE, et al. Exercise in the treatment of obesity: effects of four interventions on body composition, resting energy expenditure, appetite, and mood. *Journal of Consulting & Clinical Psychology* 1997;65(2):269-77.
46. Wadden TA, Vogt RA, Foster GD, et al. Exercise and the maintenance of weight loss: 1-year follow-up of a controlled clinical trial. *Journal of Consulting & Clinical Psychology* 1998;66(2):429-33.
47. Wing RR, Venditti E, Jakicic JM, et al. Lifestyle intervention in overweight individuals with a family history of diabetes. *Diabetes care* 1998;21(3):350-59. doi: 10.2337/diacare.21.3.350
48. Christensen P, Frederiksen R, Bliddal H, et al. Comparison of three weight maintenance programs on cardiovascular risk, bone and vitamins in sedentary older adults. *Obesity* 2013;21(10):1982-90.
49. Van Aggel-Leijssen DP, Saris WH, Hul GB, et al. Long-term effects of low-intensity exercise training on fat metabolism in weight-reduced obese men. *Metabolism: Clinical & Experimental* 2002;51(8):1003-10.
50. Alvarez C, Ramirez-Campillo R, Martinez-Salazar C, et al. Low-Volume High-Intensity Interval Training as a Therapy for Type 2 Diabetes. *International Journal of Sports Medicine* 2016;37(9):723-29. doi: 10.1055/s-0042-104935
51. Beavers KM, Beavers DP, Martin SB, et al. Change in Bone Mineral Density During Weight Loss with Resistance Versus Aerobic Exercise Training in Older Adults. *Journals of Gerontology Series A-Biological Sciences & Medical Sciences* 2017;72(11):1582-85.
52. Benito PJ, Bermejo LM, Peinado AB, et al. Change in weight and body composition in obese subjects following a hypocaloric diet plus different training programs or physical activity recommendations. *Journal of Applied Physiology* 2015;118(8):1006-13.
53. Bertram SR, Venter I, Stewart RI. Weight loss in obese women--exercise v. dietary education. *South African Medical Journal Suid-Afrikaanse Tydskrif Vir Geneeskunde* 1990;78(1):15-8.
54. Bertz F, Winkvist A, Brekke HK. Sustainable weight loss among overweight and obese lactating women is achieved with an energy-reduced diet in line with dietary recommendations: results from the LEVA randomized controlled trial. *Journal of the Academy of Nutrition & Dietetics* 2015;115(1):78-86.
55. Fayh AP, Lopes AL, da Silva AM, et al. Effects of 5 % weight loss through diet or diet plus exercise on cardiovascular parameters of obese: a randomized clinical trial. *European journal of nutrition* 2013;52(5):1443-50. doi: 10.1007/s00394-012-0450-1
56. Moraleda BR, Morencos E, Peinado AB, et al. Can the exercise mode determine lipid profile improvements in obese patients? *Nutricion Hospitalaria* 2013;28(3):607-17. doi: 10.3305/nh.2013.28.3.6284
57. Rojo-Tirado MA, Benito PJ, Peinado AB, et al. Discriminant models to estimate the body weight loss after a six-month long diet and exercise-based intervention. *Journal of sports medicine and physical fitness* 2016;56(1-2):79-84.
58. van Gemert WA, Peeters PH, May AM, et al. Effect of diet with or without exercise on abdominal fat in postmenopausal women - a randomised trial. *BMC Public Health* 2019;19(1):174.
59. Ashutosh K, Methrotra K, Fragale-Jackson J. Effects of sustained weight loss and exercise on aerobic fitness in obese women. *Journal of Sports Medicine & Physical Fitness* 1997;37(4):252-7.
60. Davidson LE, Hudson R, Kilpatrick K, et al. Effects of Exercise Modality on Insulin Resistance and Functional Limitation in Older Adults A Randomized Controlled Trial. *Archives of Internal Medicine* 2009;169(2):122-31. doi: 10.1001/archinternmed.2008.558
61. Hovell MF, Mulvihill MM, Buono MJ, et al. Culturally tailored aerobic exercise intervention for low-income Latinas. *American Journal of Health Promotion* 2008;22(3):155-63. doi: 10.4278/ajhp.22.3.155
62. Wood PD, Stefanick ML, Dreon DM, et al. Changes in plasma lipids and lipoproteins in overweight men during weight loss through dieting as compared with exercise. *New England Journal of Medicine* 1988;319(18):1173-9.
63. Ard JD, Gower B, Hunter G, et al. Effects of Calorie Restriction in Obese Older Adults: The CROSSROADS Randomized Controlled Trial. *Journals of Gerontology Series A-Biological Sciences & Medical Sciences* 2017;73(1):73-80.
64. Berg A, Berg A, Frey I, et al. Exercise based lifestyle intervention in obese adults: results of the intervention study m.o.B.I.L.I.s. *Deutsches Arzteblatt International* 2008;105(11):197-203.
65. Catenacci VA, Ostendorf DM, Pan Z, et al. The Impact of Timing of Exercise Initiation on Weight Loss: An 18-Month Randomized Clinical Trial. *Obesity* 2019;27(11):1828-38.
66. Donnelly JE, Jacobsen DJ, Heelan KS, et al. The effects of 18 months of intermittent vs continuous exercise on aerobic capacity, body weight and composition, and metabolic fitness in previously sedentary, moderately obese females. *International Journal of Obesity* 2000;24(5):566-72. doi: 10.1038/sj.ijo.0801198
67. Dutheil F, Lac G, Lesourd B, et al. Different modalities of exercise to reduce visceral fat mass and cardiovascular risk in metabolic syndrome: the RESOLVE* randomized trial. *International Journal of Cardiology* 2013;168(4):3634-42. doi: 10.1016/j.ijcard.2013.05.012
68. Jakicic JM, Marcus BH, Gallagher KI, et al. Effect of exercise duration and intensity on weight loss in overweight, sedentary women: a randomized trial. *Jama* 2003;290(10):1323-30.

69. Jakicic JM, Rickman AD, Lang W, et al. Time-based physical activity interventions for weight loss: a randomized trial. *Medicine & Science in Sports & Exercise* 2015;47(5):1061-9.
70. Muollo V, Rossi AP, Milanese C, et al. The effects of exercise and diet program in overweight people - Nordic walking versus walking. *Clinical Interventions in Aging* 2019;14:1555-65. doi: 10.2147/cia.S217570
71. Slentz CA, Bateman LA, Willis LH, et al. Effects of exercise training alone vs a combined exercise and nutritional lifestyle intervention on glucose homeostasis in prediabetic individuals: a randomised controlled trial. *Diabetologia* 2016;59(10):2088-98. doi: 10.1007/s00125-016-4051-z
72. Yassine HN, Marchetti CM, Krishnan RK, et al. Effects of exercise and caloric restriction on insulin resistance and cardiometabolic risk factors in older obese adults--a randomized clinical trial. *Journals of Gerontology Series A-Biological Sciences & Medical Sciences* 2009;64(1):90-5.
73. Vissers D, Verrijken A, Mertens I, et al. Effect of long-term whole body vibration training on visceral adipose tissue: a preliminary report. *Obes Facts* 2010;3(2):93-100. doi: 10.1159/000301785 [published Online First: 2010/05/21]
74. Alves JG, Gale CR, Mutrie N, et al. A 6-month exercise intervention among inactive and overweight favela-residing women in Brazil: the Caranguejo Exercise Trial. *American Journal of Public Health* 2009;99(1):76-80.
75. Anderssen SA, Carroll S, Urdal P, et al. Combined diet and exercise intervention reverses the metabolic syndrome in middle-aged males: results from the Oslo Diet and Exercise Study. *Scandinavian Journal of Medicine & Science in Sports* 2007;17(6):687-95. doi: 10.1111/j.1600-0838.2006.00631.x
76. Andreou E, Philippou C, Papandreou D. Effects of an intervention and maintenance weight loss diet with and without exercise on anthropometric indices in overweight and obese healthy women. *Annals of Nutrition & Metabolism* 2011;59(2-4):187-92.
77. Auerbach P, Nordby P, Bendtsen LQ, et al. Differential effects of endurance training and weight loss on plasma adiponectin multimers and adipose tissue macrophages in younger, moderately overweight men. *American Journal of Physiology - Regulatory Integrative & Comparative Physiology* 2013;305(5):R490-8.
78. Batrakoulis A, Jamurtas AZ, Georgakouli K, et al. High intensity, circuit-type integrated neuromuscular training alters energy balance and reduces body mass and fat in obese women: A 10-month training-detraining randomized controlled trial. *Plos One* 2018;13(8) doi: 10.1371/journal.pone.0202390
79. Batrakoulis A, Loules G, Georgakouli K, et al. High-intensity interval neuromuscular training promotes exercise behavioral regulation, adherence and weight loss in inactive obese women. *European Journal of Sport Science EJSS : Official Journal of the European College of Sport Science* 2019:1-10.
80. Borg P, Kukkonen-Harjula K, Fogelholm M, et al. Effects of walking or resistance training on weight loss maintenance in obese, middle-aged men: a randomized trial. *International Journal of Obesity & Related Metabolic Disorders: Journal of the International Association for the Study of Obesity* 2002;26(5):676-83.
81. Brosseau L, Wells GA, Kenny GP, et al. The implementation of a community-based aerobic walking program for mild to moderate knee osteoarthritis: A knowledge translation randomized controlled trial: Part II: Clinical outcomes. *Bmc Public Health* 2012;12 doi: 10.1186/1471-2458-12-1073
82. Campbell KL, Campbell PT, Ulrich CM, et al. No reduction in C-reactive protein following a 12-month randomized controlled trial of exercise in men and women. *Cancer Epidemiology Biomarkers & Prevention* 2008;17(7):1714-18. doi: 10.1158/1055-9965.Epi-08-0088
83. Crouse SF, Obrien BC, Grandjean PW, et al. Training intensity, blood lipids, and apolipoproteins in men with high cholesterol. *Journal of Applied Physiology* 1997;82(1):270-77.
84. de Jonge L, Moreira EA, Martin CK, et al. Impact of 6-month caloric restriction on autonomic nervous system activity in healthy, overweight, individuals. *Obesity (silver spring, md)* 2010;18(2):414-16. doi: 10.1038/oby.2009.408
85. Donnelly JE, Hill JO, Jacobsen DJ, et al. Effects of a 16-month randomized controlled exercise trial on body weight and composition in young, overweight men and women: the Midwest Exercise Trial. *Archives of internal medicine* 2003;163(11):1343-50.
86. Duncan GE, Perri MG, Anton SD, et al. Effects of exercise on emerging and traditional cardiovascular risk factors. *Preventive Medicine* 2004;39(5):894-902. doi: 10.1016/j.ypmed.2004.03.012
87. Fogelholm M, Kukkonen-Harjula K, Nenonen A, et al. Effects of walking training on weight maintenance after a very-low-energy diet in premenopausal obese women: a randomized controlled trial. *Archives of internal medicine* 2000;160(14):2177-84.
88. Friedenreich CM, Neilson HK, O'Reilly R, et al. Effects of a High vs Moderate Volume of Aerobic Exercise on Adiposity Outcomes in Postmenopausal Women: A Randomized Clinical Trial. *JAMA Oncology* 2015;1(6):766-76.
89. Hunter GR, Brock DW, Byrne NM, et al. Exercise Training Prevents Regain of Visceral Fat for 1 Year Following Weight Loss. *Obesity* 2010;18(4):690-95. doi: 10.1038/oby.2009.316
90. Womack CJ, Harris DL, Katzel LI, et al. Weight loss, not aerobic exercise, improves pulmonary function in older obese men. *Journals of Gerontology Series A-Biological Sciences & Medical Sciences* 2000;55(8):M453-7.
91. Dengel DR, Hagberg JM, Coon PJ, et al. Effects of weight loss by diet alone or combined with aerobic exercise on body composition in older obese men. *Metabolism: Clinical & Experimental* 1994;43(7):867-71.
92. Fox AA, Thompson JL, Butterfield GE, et al. Effects of diet and exercise on common cardiovascular disease risk factors in moderately obese older women. *American Journal of Clinical Nutrition* 1996;63(2):225-33.
93. Serra MC, Blumenthal JB, Ryan AS. Impact of Weight Loss and Aerobic Exercise on Nutrition and Bone Mineral Density in African American and Caucasian Postmenopausal Women. *Jarcp* 2013;2(1):11-16.

94. Beavers KM, Beavers DP, Nesbit BA, et al. Effect of an 18-month physical activity and weight loss intervention on body composition in overweight and obese older adults. *Obesity* 2014;22(2):325-31.
95. Beavers KM, Ambrosius WT, Rejeski WJ, et al. Effect of Exercise Type During Intentional Weight Loss on Body Composition in Older Adults with Obesity. *Obesity* 2017;25(11):1823-29.
96. Bell GJ, Harber V, Murray T, et al. A comparison of fitness training to a pedometer-based walking program matched for total energy cost. *Journal of physical activity & health* 2010;7(2):203-13. doi: 10.1123/jpah.7.2.203 [published Online First: 2010/05/21]
97. Dash C, Taylor TR, Makambi KH, et al. Effect of Exercise on Metabolic Syndrome in Black Women by Family History and Predicted Risk of Breast Cancer: The FIERCE Study. *Cancer* 2018;124(16):3355-63. doi: 10.1002/cncr.31569
98. Weinstock RS, Dai H, Wadden TA. Diet and exercise in the treatment of obesity: effects of 3 interventions on insulin resistance. *Archives of Internal Medicine* 1998;158(22):2477-83.
99. Gram AS, Bladbjerg EM, Quist JS, et al. Anti-inflammatory effects of active commuting and leisure time exercise in overweight and obese women and men: A randomized controlled trial. *Atherosclerosis* 2017;265:318-24. doi: 10.1016/j.atherosclerosis.2017.06.923
100. Blumenthal JA, Sherwood A, Gullette EC, et al. Exercise and weight loss reduce blood pressure in men and women with mild hypertension: effects on cardiovascular, metabolic, and hemodynamic functioning. *Archives of Internal Medicine* 2000;160(13):1947-58.
101. Lakhdar N, Denguezli M, Zaouali M, et al. Diet and diet combined with chronic aerobic exercise decreases body fat mass and alters plasma and adipose tissue inflammatory markers in obese women. *Inflammation* 2013;36(6):1239-47.
102. Messier SP, Mihalko SL, Legault C, et al. Effects of intensive diet and exercise on knee joint loads, inflammation, and clinical outcomes among overweight and obese adults with knee osteoarthritis: the IDEA randomized clinical trial. *Jama* 2013;310(12):1263-73.
103. Watkins LL, Sherwood A, Feinglos M, et al. Effects of exercise and weight loss on cardiac risk factors associated with syndrome X. *Arch Intern Med* 2003;163(16):1889-95. doi: 10.1001/archinte.163.16.1889 [published Online First: 2003/09/10]
104. Donnelly JE, Honas JJ, Smith BK, et al. Aerobic exercise alone results in clinically significant weight loss for men and women: midwest exercise trial 2. *Obesity* 2013;21(3):E219-28.
105. Wood PD, Stefanick ML, Williams PT, et al. The effects on plasma lipoproteins of a prudent weight-reducing diet, with or without exercise, in overweight men and women. *New England Journal of Medicine* 1991;325(7):461-6.

Online Supporting Information

Table S5. Characteristics of participants and training protocols in 32 randomized controlled trials included in the current systematic review.

Author, Year	Country	Study length (months)	Sample size, Female (%)	Mean age (years)	Mean BMI (kg/m²)	Specification of studied groups and exercise programs				Co-interventions in all included arms	Outcomes
						Arm 1	Arm 2	Arm 3	Arm 4		
Arsenault et al. 2009 Church et al. 2007	US	6 mo	464 100%	57.3 y	31.8	AET: supervised semi-recumbent ergometer or treadmill exercise aiming to achieve EE of (1) 4, (2) 8 or (3) 12 kcal/kg/wk (D: depending on the target EE, F: 3-4 d/wk, I: 50% $VO2_{peak}$)	C: maintain usually exercise habits	-	-	None	BW, BMI, WC, FM
Beavers et al. 2017	US	18 mo	163 71%	66.6 y	34.7	RT: resistance training on eight machines with initial resistance determined from 1 RM. Resistance was increased after each two consecutive days of sessions (D: 45 min, 3 sets of 10-12 reps, F: 4 d/wk, I: progressive to 75% RM)	C: no formal exercise	-	-	Behaviour-based weight loss program (group sessions); Diet (%E: Protein 20-25%, Fat 20-30%, CHO 45-45%)	BW, FM, FFM
Bell et al. 2010	Canada	6 mo	142 NA	49.0 y	30.5	AET: fitness training program using a treadmill, and sometimes stationary stair climbing (D: increasing from 20 to 43 min, F: from 3 to 4 d/wk, I: increasing from 55 to 70% $VO2_{peak}$)	C: maintain usual exercise habits	-	-	None	BW, BMI, WC
Blumenthal et al. 2000	US	6 mo	78 51%	46.9 y	32.7	AET: aerobic exercise sessions included 10 min warm-up, 35 min cycle ergometer and walking (eventually jogging) and 10 min cool-down exercises (D: ~55 min, F: 3-4 d/wk, I: 70-85% HRR),	C: maintain usual dietary and exercise habits	-	-	None	BW, BMI, FM, FFM

Online Supporting Information

Author, Year	Country	Study length (months)	Sample size, Female (%)	Mean age (years)	Mean BMI (kg/m²)	Specification of studied groups and exercise programs				Co-interventions in all included arms	Outcomes
						Arm 1	Arm 2	Arm 3	Arm 4		
Bouchonville et al. 2014 Villareal et al. 2011	US	12 mo	107 62.6%	69.7 y	37.2	CT: supervised group-training sessions (D: total 90 min, F:3d/wk) including aerobic exercise (D: 30 min, I: gradually increased up to 70-85% HR_{max}), resistance training on weight lifting machines (D: 30 min, 2-3 sets of 6-8 reps, I: gradually increased to 80% 1 RM), flexibility and balance exercises	C: maintain usually exercise habits, any diet or exercise programs	-	-	Hypocaloric balanced diet (500-700 kcal/d deficit and ~1 g/kg BW/d of high-quality protein)	BW, BMI, WC, FM, FFM,
Brochu et al. 2009	Canada	6 mo	137 100%	57.7 y	32.3	RT: resistance training for major muscle groups of the body divided in 4 phases (with 10 min low intensity aerobic warm-up) (D: 2-4 sets of 8-15 reps, F: 3 d/wk, I: individually monitored)	C: maintain usually exercise habits	-	-	Hypocaloric balanced diet (500-800 kcal/d deficit; %E: Protein 15%, Fat 30%, CHO 55%); Nutrition classes	BW, BMI, WC, FM, FFM
Choo et al. 2014	South Korea	9 mo	110 100%	43.1 y	28.5	RT: resistance training for major upper and lower body exercises (D: 2 sets of 8-12 reps, F: 3 d/wk, I: increasing by 5% every 3 wk from 40-60% of maximum strength)	AET: aerobic session of treadmill and bike exercise (D: 60 min, F: 3 d/wk, I: 50-70% HRR)	CT: combination of AET and RT protocols		Diet targeted to ~1200-1500 kcal and ≤25% E from fat , behavioural counselling	BW, WC, FFM
Dash et al. 2018	US	6 mo	144 100%	58.2 y	35.5	AET: supervised facility sessions including treadmill or exercise bike (D: 50 min, F: 3 d/wk, I: 45-65% $VO2_{peak}$)	C: maintain usually exercise habits	-	-	None	WC
Davidson et al. 2009	Canada	6 mo	136 58%	67.6 y	30.0	AET: treadmill walking (D: ~30 min, F: 5 d/wk, I: 60-75% $VO2_{peak}$),	RT: resistance training for nine major muscle groups (D: ~20 min, 1 set of 15 reps, F: 3 d/wk, I: progressive),	CT: combination of RT and AET protocols	C: no exercise	Nutrition seminars	BW, BMI, WC, FM
Donnelly et al. 2013	US	10 mo	63 0%	23.4 y	31.7	AET(1+2): supervised sessions of treadmill walking or jogging aiming to achieve EE of (1) 400 or (2) 600 kcal/session (D: depending on the target EE, F: 5 d/wk, I: 70 progressive to 80% HR_{max})	C: no exercise	-	-	None	BW, BMI, WC, FM, FFM

Online Supporting Information

Author, Year	Country	Study length (months)	Sample size, Female (%)	Mean age (years)	Mean BMI (kg/m^2)	Specification of studied groups and exercise programs				Co-interventions in all included arms	Outcomes
						Arm 1	Arm 2	Arm 3	Arm 4		
Foster-Schubert et al. 2012	US	12 mo	439<br>100%	58.0 y	30.9	AET: facility supervised (treadmill walking, stationary bicycling and other aerobic machines) and home (walking/hiking, aerobics, bicycling) exercise sessions (D: ~45 min, F: 5 d/wk, I: progressed to 70-85% HR_{max}),	C: exercise habits not changed.	-	-	Diet (1200-2000 kcal/d - based on body weight, %E: Fat <30%); Additional dietary counselling sessions	BW, BMI, WC, FM, FFM,
Frimel et al. 2008	US	6 mo	30<br>60%	69.5 y	36.8	CT: exercise training session (D: 90 min, F: 3d/wk), of flexibility exercises, low-impact aerobic exercises and high-intensity resistance training of nine exercises (D: increasing to 3 sets of 8-12 reps, I: 85% 1 RM)	C: no exercise	-	-	Hypocaloric balanced diet (~750 kcal/d deficit, %E: Protein 20%, Fat 30%, CHO 50%); Behavioural strategies for better eating habits	BW, FM, FFM
Gepner et al. 2017	Izrael	12 mo	278<br>11%	47.8 y	30.8	CT: aerobic training combined with resistance training (D: increasing to 45 min (AET)/15 min (RT), F: 3 d/wk, I: (AET) increasing to 80% HR_{max} and (RT) 2 sets with weight of 80% maximum weight)	C: no added physical activity	-	-	Equal-caloric MedDiet/ low-CHO (CHO intake <40-70 g/d, MedDiet food items) or low-fat diet (%E: Fat ≤30%, SFA <10%);<br>Nutritional session and educational workshops	BW, BMI, WC
Gram et al. 2017	Denmark	6 mo	56<br>54.7%	35.6 y	30.1	AET: vigorous intensity (D/F: aiming to achieve EE of 1600 kcal/week for women and 2100 kcal for men, I: ~70% $VO2_{peak}$)	C: maintain habitual lifestyle	-	-	None	BW, BMI, WC, FM, FFM,
Irwin et al. 2003	US	12 mo	173<br>100%	60.8 y	30.6	CT: combination of overall moderate-intensity exercises (D: 45 min, F: 5 d/wk, I: gradually increased to 60-75% HR_{max}) from which one was facility session (treadmill, stationary bicycling, strength training of 2 sets of 10 reps for major muscle groups) and four sessions of various home exercises.	MI: attended to stretching sessions (D: 45 min, F: 1 d/wk) and not changed other exercise habits.	-	-	Group exercise behaviour-change education classes	BW, BMI, WC, FM

Online Supporting Information

Author, Year	Country	Study length (months)	Sample size, Female (%)	Mean age (years)	Mean BMI (kg/m²)	Specification of studied groups and exercise programs				Co-interventions in all included arms	Outcomes
						Arm 1	Arm 2	Arm 3	Arm 4		
Lakhadar et al. 2013	Tunisia	6 mo	20<br>100%	38.9 y	33.0	AET: treadmill walking or running (D: gradually increased to 45 min, F: 3 d/wk, I: gradually increased to 80% HR_{max}),	C: no added physical activity	-	-	Balanced, personalized hypocaloric diet (500 kcal deficit, %E: Protein 15%, Fat 30%, CHO 55%);	BW, BMI, WC, FM
Martin et al. 2019	US	6 mo	198<br>72.5%	48.9 y	31.5	AET(1+2): monitored/ supervised treadmill training with altered speed and grade, which allowed to keep the targeted HR range (F: participants-selected 3-5 d/wk, I: 65-85% $VO2_{peak}$) and duration reflecting (1) general health (D: 8 kcal/kg body weight/week, about 800-1000 kcal/wk) or (2) weight loss (D: 20 kcal/kg body weight/week, about 2000 – 2250 kcal/wk);	MI: maintained baseline physical activity level and healthy lifestyle information seminars	-	-	None	BW, BMI, WC, FM, FFM
Messier et al. 2013	US	18 mo	304<br>71.4%	65.5 y	33.7	CT: training protocol includes four phases: aerobic (15 min), resistance training (20min), aerobic (15 min), cool-down (10 min). Aerobic training involved walking or alternatively stationary bicycling. Resistance training consisted of exercises for upper and lower body parts. (D: 60 min, F: 3 d/wk, I: 50-75% HRR);	C: no added physical activity	-	-	Hypocaloric diet (deficit up to 1100 kcal for women and 1200 kcal for men, %E: Protein 15-20%, Fat <30%, CHO 45-60%);<br>Nutrition education and behavioural sessions	BW, FM, FFM
Nicklas et al. 2004	US	18 mo	316<br>72%	68.5 y	34.4	CT: training protocol includes four phases: aerobic, resistance training, aerobic, cool-down, 15 min each (D: 60 min including 2 sets of 12 reps, F: 3 d/wk, I:50-75% HRR and increasing weight);	MI: health education and social interaction	-	-	Three-phases (intensive, transition, maintenance) dietary weight-loss intervention (deficit by 500 kcal/d), including individual and group sessions about eating behaviour	BW, BMI

Online Supporting Information

Author, Year	Country	Study length (months)	Sample size, Female (%)	Mean age (years)	Mean BMI (kg/m²)	Specification of studied groups and exercise programs				Co-interventions in all included arms	Outcomes
						Arm 1	Arm 2	Arm 3	Arm 4		
Park et al. 2017	South Korea	6 mo	50<br>100%	74.1 y	27.3	CT: resistance training with elastic band use for 12 body parts (D: 20-30 min, F: 5d/wk, I: 2-3 sets, each 8-15 reps) and aerobic exercise of various walking activities (D: 30-50 min, F: 5d/wk, I: 13-17 RPE) with 10 min warm-up and cool-down;	C: maintained usual physical activity	-	-	None	WC
Potteiger et al. 2012	US	6 mo	35<br>0%	36.1 y	31.2	RT: resistance training for all major muscle groups, training loads changed within each week (D: 45 min, 4 sets of 5-10 reps, F: 4 d/wk, I: changing: 5-7 reps with 100% of 5-7 RM or 8-10 reps with 80% of 8-10 RM)	AET: treadmill walking or jogging, stationary cycling and stationary stair climbing (D: 45 min, F: 4 d/wk, I: 65-80% HR_{max}).	-	-	Hypocaloric diet (300-600 kcal/d deficit) achieved by lower portions and choosing low-fat products	BW, BMI, WC, FM, FFM,
Ross et al. 2015	Canada	6 mo	300<br>65.3%	51.4 y	33.4	AET: (1) low-, low-, (2) low-, high-, (3) high-amount and how-intensity treadmill training (D: EE target of (1,2) 180-300 kcal or (3) 360-600 kcal, F: 5 d/wk, I: (1) 50% $VO2_{peak}$ or (2,3) 75% $VO2_{peak}$)	C: maintain usually exercise habits.	-	-	None	BW, BMI, WC
Said et al. 2017	Tunisia	6 mo	32<br>100%	30.1 y	32.6	CT: sessions (F:4 d/wk) of rhythmic aerobic exercises without jumping (D: 30 min, I: increasing to 60-65 HR_{max}), muscle strengthening exercises on machines for seven major muscle groups (D: 20 min, 2 sets, I: increasing to 80% 1 RM), warm-up and cool down	AET: sessions of high-impact aerobic rhythmic exercises in which both feet leave the ground with warm-up and cool-down (D: 50-60 min, F: 4 d/wk, I: increasing to 85% HR_{max});	-	-	None	BW, BMI, WC, FM, FFM,

Online Supporting Information

Author, Year	Country	Study length (months)	Sample size, Female (%)	Mean age (years)	Mean BMI (kg/m²)	Specification of studied groups and exercise programs				Co-interventions in all included arms	Outcomes
						Arm 1	Arm 2	Arm 3	Arm 4		
Shah et al. 2009	US	6 mo	18<br>72.2%	68.6 y	≥30	CT: training combined of 15 min flexibility exercises, 30 min aerobic exercises, 30 min strength training and 15 min balance exercises (D:~90 min, F: 3 d/wk, I: (AET) gradually increased to ~85% HR_{peak} and (RM) 2-3 set of 6-8 reps with ~80 1 RM%)	C: recommendations for exercise were not specified;	-	-	Hypocaloric balanced diet (500-1000 kcal/d deficit, %E: Protein 20%, Fat 30%, CHO 50%); behavioral education	BW, FM, FFM
Slentz et al. 2011<br>Willis et al. 2012	US	32 wk	196<br>56.3%	48.8 y	30.5	AET: aerobic training with the use of treadmill, elliptical trainers, cycle ergometers or its combination (D: ~19.2 km/wk, about 132 min/wk, F: 3 d/wk, I:75% $VO2_{peak}$);	RT: supervising weight lifting sessions on eight machines for major muscle groups, (D: 3 sets of 8-12 reps, F:3 d/wk, I: progressive weight lifted);	CT: combination of AET and RT.	-	None	BW, WC, FM, FFM
Sweeney et al. 1993	US	6 mo	47<br>100%	34.2 y	35.1	AET: 15 min warm-up and aerobic training of steadily increased distance of indoor track brisk walking (increasing to D: 8.8 km, F: 3 d/wk, I: 24-27 min/km with 70-85% HR_{max})	CT: same as AET (increasing to D: 6.8 km/session, F: 3 d/wk, I: 70-85% THR) plus circuit weight training of all muscle groups on seven weight and one sit-up stations with 30 s each (D: ≈5 min 45 s, F: 3 d/wk, I: increasing to 40% 1 RM)	C: no added physical activity	-	Hypocaloric diet (with %E: Protein≈15%, Fat≈30%, CHO≈55%) with two E targets:<br>(1) Severe energy restriction providing 40% E requirement<br>(2) moderate energy restriction providing 70%E requirement	BW, FM
Villareal et al. 2017	US	6 mo	120<br>64.4%	70.0 y	36.4	AET: training sessions included flexibility, 40 min aerobic (treadmill walking, stationary cycling, stairs climbing) and balance exercises (D: 60 min, F: 3d/wk, I:gradually increased from 65 to 70-85% HR_{peak})	RT: training sessions included flexibility, 40 min resistance training (nine lower- and upper-body exercises on weight-lifting machines) and balance exercises (D: 60 min, 2-3 sets of 8-12 reps, F: 3 d/wk, I: gradually increased to 85% 1 RM)	CT: training sessions included flexibility, 30-40 min aerobic, 30-40 min resistance and balance exercises (D: 75-90 min, F: 3 d/wk, I: like in AET/RT protocols)	-	Weight-management program. Balanced hypocaloric diet (500 – 750 kcal/d deficit and ~1 g/kg BW/d of high-quality protein).	BW, BMI, FM, FFM
Watkins et al. 2003	US	6 mo	32<br>NA	≥ 29 y	33.1	AET: supervised exercise sessions of cycle ergometry or walking, eventually jogging, each with warm -up and cool-down (D: 35 min, F: 3 to 4 d/wk, I: 70-80% of initial HRR);	C: maintain usually exercise habits	-	-	None	BW, BMI, WC, FM

Online Supporting Information

Author, Year	Country	Study length (months)	Sample size, Female (%)	Mean age (years)	Mean BMI (kg/m²)	Specification of studied groups and exercise programs				Co-interventions in all included arms	Outcomes
						Arm 1	Arm 2	Arm 3	Arm 4		
Weinstock et al. 1998	US	12 mo	31<br>100%	43.3 y	35.7	RT: resistance training targeting the large muscle groups with 5-10 min warm-up and 5 min cool-down (increasing up to D: 40 min, 2 sets, F: 3 d/wk by 28 wk and 2 d/wk at 29 - 48 wk, I: resistance allowed to do 10-14 repetitions by wk 14)	C: no regular or formal exercise	-	-	Hypocaloric diet (from 925kcal/d at week 1 to 1500 kcal with %E: Protein 12-15%, Fat 25-30%, CHO 55-60% beginning at week 22)	BW, BMI
Wood et al. 1991	US	12 mo	89<br>0%	40.3 y	30.7	AET: brisk walking or jogging (D: from 25 min initially gradually to 45 min, F: 3d/wk, I: 60-80% HR_{max});	C: no change in exercise level	-	-	Hypocaloric NCPE diet (%E: Protein 15%, Fat 30%, CHO 55 %)	BW, FM
You et al. 2004	US	6 mo	50<br>100%	58.0 y	32.8	AET: treadmill walking (progressed from the first week to the third month D: from 30 to 45-60 min, F:3d/wk, I: 50-55% to 65-70% HRR)	C: no formal exercise	-	-	Hypocaloric diet (~250-350 kcal/d deficit); Dietary instructions	BW, FM, FFM
Zhang et al. 2016	China	12 mo	220<br>67.7%	53.9 y	28.0	AET: **(1)** brisk walking (D: 30 min, F: 5 d/wk for 48 wk, I: 45-55% HR) or **(2)** supervised treadmill jogging (D: 30 min, F: 5 d/wk for the first 28 wk, I: 65-80% HR) and brisk walking (D: 30 min, F: 5 d/wk starting from 28 wk, I: 45-55% HR_{max});	C: not change in physical activity	-	-	Health education sessions (identical, separately for each study group)	BW, WC, FM

Training modalities: AET – aerobic training, C – control group, CT – combined training, MI – minimal intervention, RT – resistance training;
Exercise prescription: D – duration of each exercise session, F – exercise frequency, I – exercise intensity; **Aerobic training protocols:** EE – energy expenditure, HR_{peak} and $_{max}$ – peak and maximum heart rate, HRR – heart rate reserve, RPE – Borg Scale of Perceived Exertion, $VO2_{peak}$ and $_{max}$ – peak and maximal oxygen consumption; **Resistance training protocols:** reps – repetitions, RM – repetition maximum;
Outcomes: BMI – body mass index, BW – body weight, FFM – free fat mass, FM – fat mass, WC – waist circumference;
Co-interventions: CHO – carbohydrates, %E – percentage of energy intake, MedDiet – Mediterranean diet, NCPE – National Cholesterol Education Program, SFA – saturated fatty acid;

Online Supporting Information

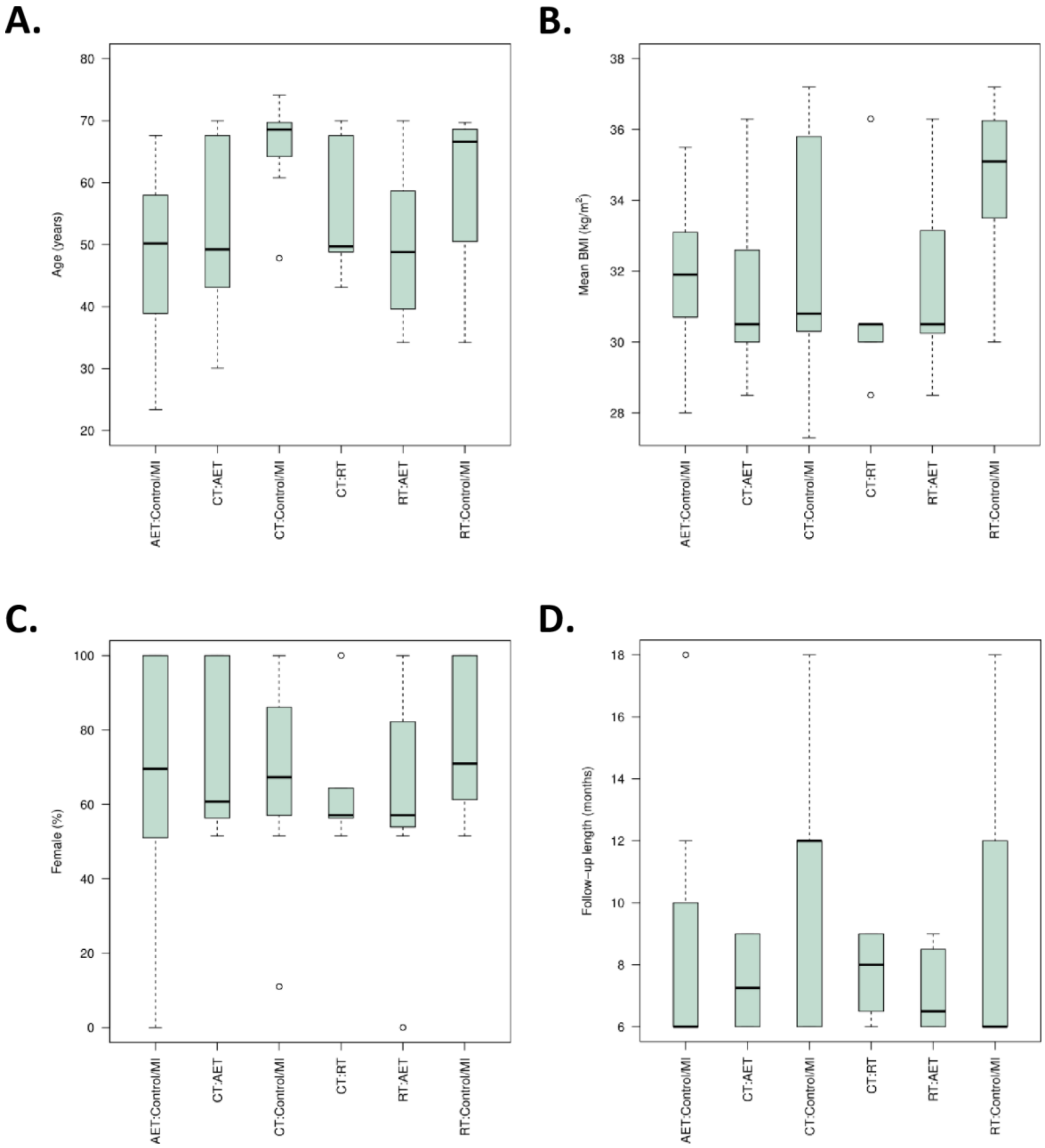

Figure S1. Boxplots presenting the distribution of (**A**) age, (**B**) body mass index (BMI), (**C**) % of female, and (**D**) follow-up length for different pairs of comparisons.

Online Supporting Information

Risk of bias domains

Study	D1	D2	D3	D4	D5	Overall
Arsenault 2009	+	+	X	+	+	X
Beavers 2017	-	-	X	+	+	X
Bell 2010	-	-	X	+	+	X
Blumentahl 2000	-	-	+	-	-	-
Bouchonville 2014	+	+	+	+	+	+
Brochu 2009	-	-	X	+	+	X
Choo 2014	+	-	-	+	-	-
Church 2007	+	+	+	+	+	+
Dash 2018	+	-	X	+	-	X
Davidson 2009	-	+	+	+	+	-
Donnelly, 2013	+	-	+	+	+	-
Foster-Schubert 2010	+	+	+	+	+	+
Frimel 2008	-	-	+	+	-	-
Gepner 2018	+	+	+	+	+	+
Gram 2017	-	X	+	+	+	X
Irwin 2003	+	+	+	+	+	+
Lakhdar 2013	-	-	+	-	-	-
Martin 2019	+	-	+	+	+	-
Messier 2013	+	-	+	+	-	-
Nicklas 2004	+	-	-	+	-	-
Park 2017	-	+	+	+	-	-
Potteiger 2012	-	-	X	+	+	X
Ross 2015	+	+	+	+	+	+
Said 2015	-	-	+	+	+	-

Online Supporting Information

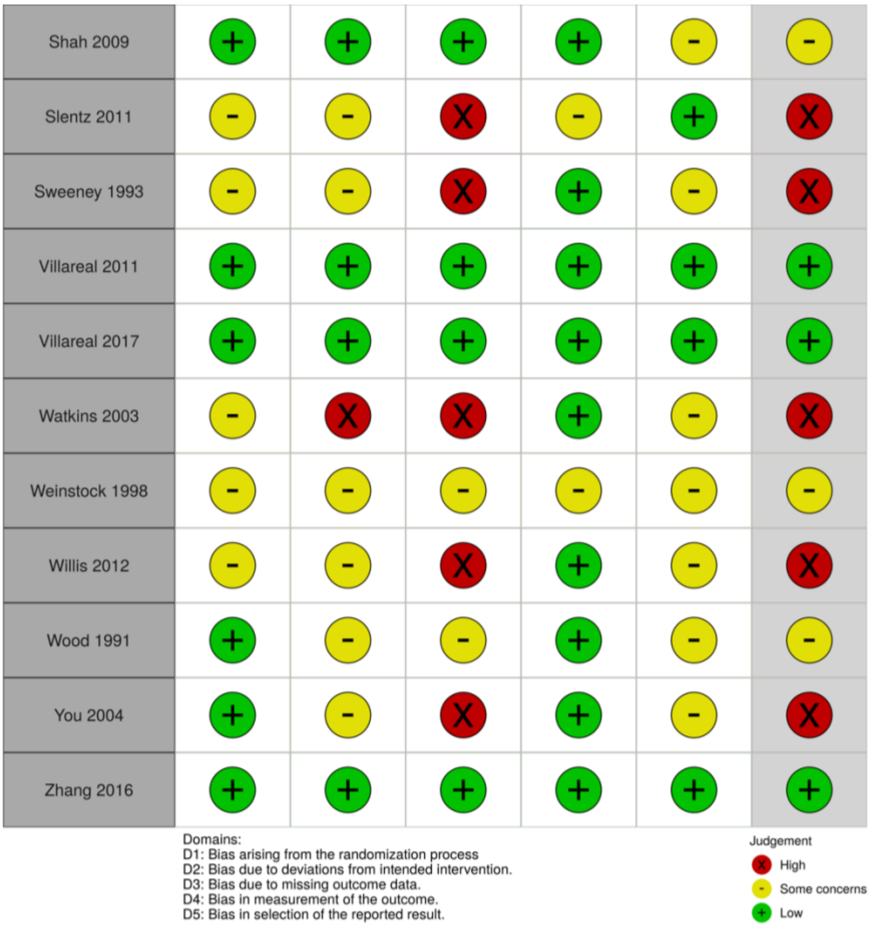

Figure S2. Summary of the risk of bias assessment for studies included in the current review.

Table S6. GRADE evaluation for body weight (kg) and all comparisons.*

	Direct evidence			Indirect evidence		Network meta-analysis	
**Comparison**	**N studies**	**MD (95% CI)**	**Certainty of evidence**	**MD (95% CI)**	**Certainty of evidence**	**MD (95% CI)**	**Certainty of evidence**
AET vs. Control/MI	16	-2.32 [-3.11; -1.53]	⊕⊕⊕◯ [1]	-1.55 [-3.27; 0.17]	⊕⊕⊕◯	-2.18 [-2.90; -1.46]	⊕⊕⊕◯ [3,4]
AET vs. CT	5	-0.34 [-1.62; 0.94]	⊕⊕⊕⊕	-1.61 [-3.12; -0.10]	-	-0.87 [-1.85; 0.10]	⊕⊕⊕◯ [3,4,5]
AET vs. RT	6	-1.87 [-3.13; -0.62]	⊕⊕⊕◯ [2]	-1.44 [-3.24; 0.36]	⊕⊕⊕◯	-1.73 [-2.76; -0.70]	⊕⊕◯◯ [3,4,5]
CT vs. Control/MI	6	-0.95 [-2.06; 0.15]	⊕⊕⊕◯ [1]	-2.02 [-3.58; -0.45]	⊕⊕⊕◯	-1.31 [-2.21; -0.40]	⊕⊕◯◯ [3,4,5]
RT vs. Control/MI	6	-1.09 [-2.45; 0.27]	⊕⊕⊕◯ [2]	0.30 [-1.18; 1.78]	⊕⊕⊕◯	-0.45 [-1.45; 0.55]	⊕⊕◯◯ [3,4,5]
CT vs. RT	4	-1.46 [-2.77; -0.19]	⊕⊕⊕◯ [1]	0.77 [-1.30; 2.85]	⊕⊕⊕◯	-0.86 [-1.94; 0.23]	⊕⊕◯◯ [3,4,5]

AET – aerobic exercise training, CT – combined training, MI – minimal intervention, RT – resistance training, MD – mean difference, CI – confidence interval;

⊕⊕⊕⊕ High; ⊕⊕⊕◯ Moderate; ⊕⊕◯◯ Low; ⊕◯◯◯ Very low.

[1] downgraded due to inconsistency (I^2 >50%);
[2] downgraded due to risk of bias (approximately 1/3 of included RCTs rated with high risk of bias);
[3] not downgraded due to incoherence (dominant estimate similar to network estimate);
[4] direct evidence contributing more to the NMA estimate (>50%);
[5] downgraded due to imprecision (95% CI overlaps important minimal important difference: -1 kg; and/or important harm: +1 kg);

*Direct estimates were evaluated with the following GRADE criteria: risk of bias, indirectness, inconsistency and publication bias. As suggested recently by the GRADE working group, consideration of imprecision is not necessary when rating the direct and indirect estimates to inform the rating of NMA estimates.

Online Supporting Information

Table S7. GRADE evaluation for body mass index (kg/m^2) and all comparisons.*

	Direct evidence			Indirect evidence		Network meta-analysis	
**Comparison**	**N studies**	**MD (95% CI)**	**Certainty of evidence**	**MD (95% CI)**	**Certainty of evidence**	**MD (95% CI)**	**Certainty of evidence**
AET vs. Control/MI	11	-0.99 [-1.36; -0.62]	⊕⊕○○ [1,2]	-0.66 [-1.60; 0.27]	⊕⊕⊕○	-0.94 [-1.29; -0.60]	⊕⊕○○ [3,4]
AET vs. CT	3	-0.16 [-0.84; 0.53]	⊕⊕⊕⊕	-0.72 [-1.41; -0.03]	-	-0.43 [-0.92; 0.05]	⊕⊕⊕○ [3,4,5]
AET vs. RT	3	-0.54 [-1.22; 0.13]	⊕⊕⊕○ [2]	-0.41 [-1.25; 0.42]	⊕⊕○○	-0.49 [-1.02; 0.03]	⊕⊕○○ [3,4,5]
CT vs. Control/MI	4	-0.44 [-0.92; 0.55]	⊕⊕⊕○ [1]	-0.76 [-1.66; 0.14]	⊕⊕○○	-0.51 [-0.94; -0.08]	⊕⊕○○ [3,4,5]
RT vs. Control/MI	4	-0.55 [-1.15; 0.06]	⊕⊕⊕○ [1]	-0.26 [-1.11; 0.59]	⊕⊕○○	-0.45 [-0.94; 0.04]	⊕⊕○○ [3,4,5]
CT vs. RT	2	-0.31 [-1.01; 0.39]	⊕⊕⊕○ [1]	0.39 [-0.54; 1.32]	⊕⊕⊕○	-0.06 [-0.62; 0.50]	⊕⊕○○ [3,4,5]

AET – aerobic exercise training, CT – combined training, MI – minimal intervention, RT – resistance training, MD – mean difference, CI – confidence interval;

⊕⊕⊕⊕ High; ⊕⊕⊕○ Moderate; ⊕⊕○○ Low; ⊕○○○ Very low.

[1] downgraded due to inconsistency (I^2 >50%);
[2] downgraded due to risk of bias (approximately 1/3 of included RCTs rated with high risk of bias);
[3] not downgraded due to incoherence (dominant estimate similar to network estimate);
[4] direct evidence contributing more to the NMA estimate (>50%);
[5] downgraded due to imprecision (95% CI overlaps important minimal important difference: -0.5 kg/m^2; and/or important harm: +0.5 kg/m^2);

*Direct estimates were evaluated with the following GRADE criteria: risk of bias, indirectness, inconsistency and publication bias. As suggested recently by the GRADE working group, consideration of imprecision is not necessary when rating the direct and indirect estimates to inform the rating of NMA estimates.

Table S8. GRADE evaluation for waist circumference (cm) and all comparisons.*

	Direct evidence			Indirect evidence		Network meta-analysis	
**Comparison**	**N studies**	**MD (95% CI)**	**Certainty of evidence**	**MD (95% CI)**	**Certainty of evidence**	**MD (95% CI)**	**Certainty of evidence**
AET vs. Control/MI	12	-2.49 [-3.42; -1.56]	⊕⊕○○ [1,2]	-1.37 [-3.60; 0.87]	⊕⊕⊕○	-2.33 [-3.19; -1.47]	⊕⊕○○ [3,4]
AET vs. CT	4	-0.08 [-1.73; 1.57]	⊕⊕⊕⊕	-0.54 [-2.29; 1.21]	-	-0.30 [-1.50; 0.90]	⊕⊕⊕○ [3,4,5]
AET vs. RT	4	-1.42 [-3.01; 0.17]	⊕⊕⊕○ [2]	-0.61 [-3.11; 1.88]	⊕⊕○○	-1.19 [-2.53; 0.15]	⊕⊕○○ [3,4,5]
CT vs. Control/MI	5	-2.07 [-3.36; -0.79]	⊕⊕⊕○ [1]	-1.91 [-4.01; 0.19]	⊕⊕○○	-2.03 [-3.12; -0.94]	⊕⊕⊕○ [3,4,6]
RT vs. Control/MI	2	-2.16 [-4.06; -0.27]	⊕⊕⊕○ [2]	-0.14 [-2.01; 1.74]	⊕⊕○○	-1.14 [-2.47; 0.19]	⊕⊕○○ [3,5]
CT vs. RT	3	-1.31 [-2.97; 0.36]	⊕⊕⊕○ [2]	0.23 [-2.49; 2.94]	⊕⊕⊕○	-0.89 [-2.31; 0.53]	⊕⊕○○ [3,4,5]

AET – aerobic exercise training, CT – combined training, MI – minimal intervention, RT – resistance training, MD – mean difference, CI – confidence interval;

⊕⊕⊕⊕ High; ⊕⊕⊕○ Moderate; ⊕⊕○○ Low; ⊕○○○ Very low.

[1] downgraded due to inconsistency (I^2 >50%);
[2] downgraded due to risk of bias (approximately 1/3 of included RCTs rated with high risk of bias);
[3] not downgraded due to incoherence (dominant estimate similar to network estimate);
[4] direct evidence contributing more to the NMA estimate (>50%);
[5] downgraded due to imprecision (95% CI overlaps important minimal important difference: -1 cm; and/or important harm: +1 cm);
[6] not downgraded due to imprecision (value close to minimal important difference/harm);

*Direct estimates were evaluated with the following GRADE criteria: risk of bias, indirectness, inconsistency and publication bias. As suggested recently by the GRADE working group, consideration of imprecision is not necessary when rating the direct and indirect estimates to inform the rating of NMA estimates.

Table S9. GRADE evaluation for fat mass (kg) and all comparisons.*

	Direct evidence			Indirect evidence		Network meta-analysis	
**Comparison**	**N studies**	**MD (95% CI)**	**Certainty of evidence**	**MD (95% CI)**	**Certainty of evidence**	**MD (95% CI)**	**Certainty of evidence**
AET vs. Control/MI	14	-1.54 [-2.29; -0.79]	⊕⊕⊕◯ [1]	-1.56 [-3.27; 0.15]	⊕⊕◯◯	-1.54 [-2.23; -0.85]	⊕⊕⊕◯ [3,4,6]
AET vs. CT	4	0.53 [-0.75; 1.80]	⊕⊕⊕⊕	0.14 [-1.48; 1.77]	-	0.38 [-0.62; 1.38]	⊕⊕⊕◯ [3,4,5]
AET vs. RT	5	-0.79 [-2.03; 0.44]	⊕⊕◯◯ [1,2]	0.23 [-1.43; 1.89]	⊕⊕⊕◯	-0.43 [-1.42; 0.56]	⊕◯◯◯ [3,4,5]
CT vs. Control/MI	4	-1.77 [-3.02; -0.53]	⊕⊕⊕◯ [1]	-2.15 [-3.67; -0.63]	⊕⊕⊕◯	-1.92 [-2.89; -0.96]	⊕⊕⊕◯ [3,4,6]
RT vs. Control/MI	5	-1.55 [-2.80; -0.29]	⊕⊕⊕◯ [2]	-0.50 [-1.99; 0.99]	⊕⊕◯◯	-1.11 [-2.07; -0.15]	⊕⊕◯◯ [3,4,5]
CT vs. RT	3	-1.27 [-2.56; 0.01]	⊕⊕◯◯ [1,2]	0.40 [-1.68; 2.48]	⊕⊕⊕◯	-0.81 [-1.90; 0.28]	⊕◯◯◯ [3,4,5]

AET – aerobic exercise training, CT – combined training, MI – minimal intervention, RT – resistance training, MD – mean difference, CI – confidence interval;

⊕⊕⊕⊕ High; ⊕⊕⊕◯ Moderate; ⊕⊕◯◯ Low; ⊕◯◯◯ Very low.

[1] downgraded due to inconsistency (I^2 >50%);
[2] downgraded due to risk of bias (approximately 1/3 of included RCTs rated with high risk of bias);
[3] not downgraded due to incoherence (dominant estimate similar to network estimate);
[4] direct evidence contributing more to the NMA estimate (>50%);
[5] downgraded due to imprecision (95% CI overlaps important minimal important difference: -1 kg; and/or important harm: +1 kg);
[6] not downgraded due to imprecision (value close to minimal important difference/harm);

*Direct estimates were evaluated with the following GRADE criteria: risk of bias, indirectness, inconsistency and publication bias. As suggested recently by the GRADE working group, consideration of imprecision is not necessary when rating the direct and indirect estimates to inform the rating of NMA estimates.

Online Supporting Information

Table S10. GRADE evaluation for fat-free mass (kg) and all comparisons.*

	Direct evidence			Indirect evidence		Network meta-analysis	
**Comparison**	**N studies**	**MD (95% CI)**	**Certainty of evidence**	**MD (95% CI)**	**Certainty of evidence**	**MD (95% CI)**	**Certainty of evidence**
AET vs. Control/MI	7	0.13 [-0.54; 0.81]	⊕⊕⊕○ [1]	-0.22 [-1.25; 0.81]	⊕⊕○○	0.03 [-0.54; 0.59]	⊕⊕⊕○ [3,4]
AET vs. CT	4	-0.90 [-1.76; -0.04]	⊕⊕⊕⊕	-1.21 [-2.56; 0.15]	-	-0.99 [-1.71; -0.26]	⊕⊕⊕○ [3,4,5]
AET vs. RT	4	-1.32 [-2.18; -0.46]	⊕⊕⊕○ [2]	-0.45 [-1.54; 0.64]	⊕⊕○○	-0.98 [-1.66; -0.31]	⊕⊕○○ [3,4,5]
CT vs. Control/MI	2	1.70 [0.46; 2.94]	⊕⊕⊕⊕	0.64 [-0.29; 1.56]	-	1.02 [0.27; 1.76]	⊕⊕⊕○ [3,5]
RT vs. Control/MI	3	0.51 [-0.36; 1.37]	⊕⊕○○ [1,2]	1.66 [0.68; 2.64]	⊕⊕⊕○	1.01 [0.36; 1.66]	⊕○○○ [3,4,5]
CT vs. RT	3	-0.43 [-1.32; 0.45]	⊕⊕⊕○ [2]	1.17 [-0.28; 2.62]	⊕⊕⊕○	0.00 [-0.75; 0.76]	⊕⊕⊕○ [3,4]

AET – aerobic exercise training, CT – combined training, MI – minimal intervention, RT – resistance training, MD – mean difference, CI – confidence interval;

⊕⊕⊕⊕ High; ⊕⊕⊕○ Moderate; ⊕⊕○○ Low; ⊕○○○ Very low.

[1] downgraded due to inconsistency (I^2 >50%);
[2] downgraded due to risk of bias (approximately 1/3 of included RCTs rated with high risk of bias);
[3] not downgraded due to incoherence (dominant estimate similar to network estimate);
[4] direct evidence contributing more to the NMA estimate (>50%);
[5] downgraded due to imprecision (95% CI overlaps important minimal important difference: 1 kg; and/or important harm: -1 kg);

*Direct estimates were evaluated with the following GRADE criteria: risk of bias, indirectness, inconsistency and publication bias. As suggested recently by the GRADE working group, consideration of imprecision is not necessary when rating the direct and indirect estimates to inform the rating of NMA estimates.

Table S11. Relative ranking* of training effects on anthropometric outcomes.

Training modality	Body weight (↓)	BMI (↓)	Waist circumference (↓)	Fat mass (↓)	Fat-free mass (↑)	All outcomes combined
AET	**0.99**	**0.98**	**0.88**	0.68	0.18	**0.74**
RT	0.29	0.54	0.37	0.42	**0.83**	0.49
CT	0.66	0.47	0.73	**0.90**	**0.83**	0.72
Control/MI	0.06	0.02	0.02	0.00	0.16	0.05

*P-scores were calculated and presented to obtain relative ranking of training modalities. Higher P-score value indicates greater benefit (larger decrease or increase in outcome of interest) of certain intervention.

Ranking for all outcomes combined (assuming their equal importance) was obtained by taking an average of all P-scores for certain intervention.

Bolded are training modalities identified as the best for the given outcome.

AET – aerobic exercise training, CT – combined training, MI – minimal intervention, RT – resistance training;

↑ – increase is the effect of interest; ↑ – decrease is the effect of interest;

Table S12. Results of node-splitting approach to assess inconsistency for anthropometric outcomes.

Comparison	Body weight		Body mass index		Waist circumference		Fat mass		Fat-free mass	
	D	P-value	D	P-value	D	P-value	D	P-value	D	P-value
AET vs. Control/MI	-0.77	0.43	-0.33	0.52	-1.13	0.36	0.02	0.98	0.35	0.57
AET vs. CT	1.27	0.21	0.56	0.26	0.46	0.71	0.38	0.72	0.31	0.7
AET vs. RT	-0.43	0.7	-0.13	0.81	-0.81	0.59	-1.03	0.33	-0.87	0.22
CT vs. Control/MI	1.06	0.28	0.32	0.54	-0.17	0.9	0.38	0.71	1.07	0.18
RT vs. Control/MI	-1.39	0.17	-0.29	0.59	-2.02	0.14	-1.04	0.29	-1.15	0.08
CT vs. RT	-2.23	0.07	-0.7	0.24	-1.53	0.35	-1.67	0.18	-1.6	0.06

D – difference between direct and indirect estimates presented in Supplementary Tables 4-8;

AET – aerobic exercise training, CT – combined training, MI – minimal intervention, RT – resistance training;

Online Supporting Information

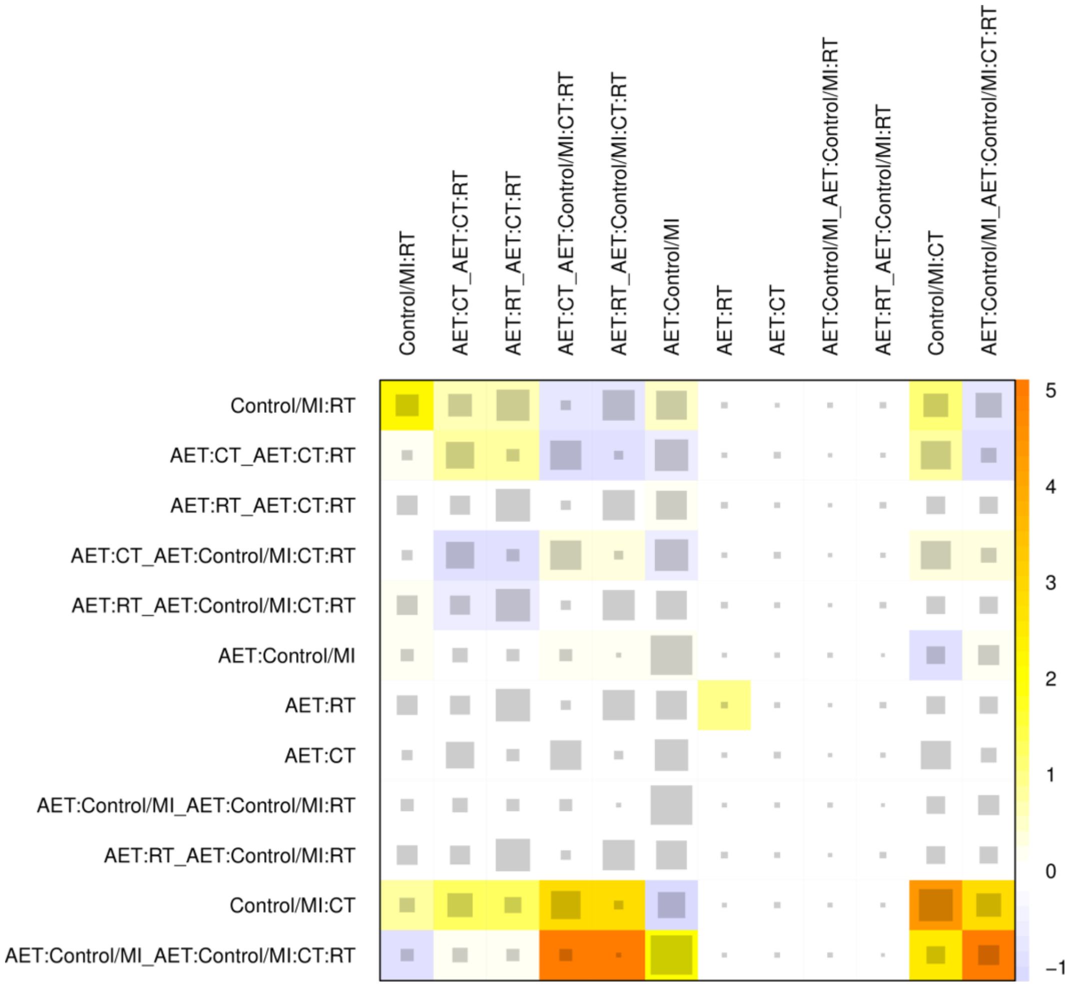

Figure S3. Net-heat plot* to assess inconsistency for body weight.

AET – aerobic exercise training, CT – combined training, MI – minimal intervention, RT – resistance training;

*This plot is a heat map where the colours on the diagonal represent the inconsistency contribution of the corresponding design and the colours on the off-diagonal are associated with the change in inconsistency between direct and indirect evidence in a network estimate in the row after relaxing the consistency assumption for the effect of a design in the column. A blue coloured element indicates that the evidence of the design in the column supports the evidence in the row; a red coloured element indicates that the evidence of the design in the column contrasts to the evidence in the row.

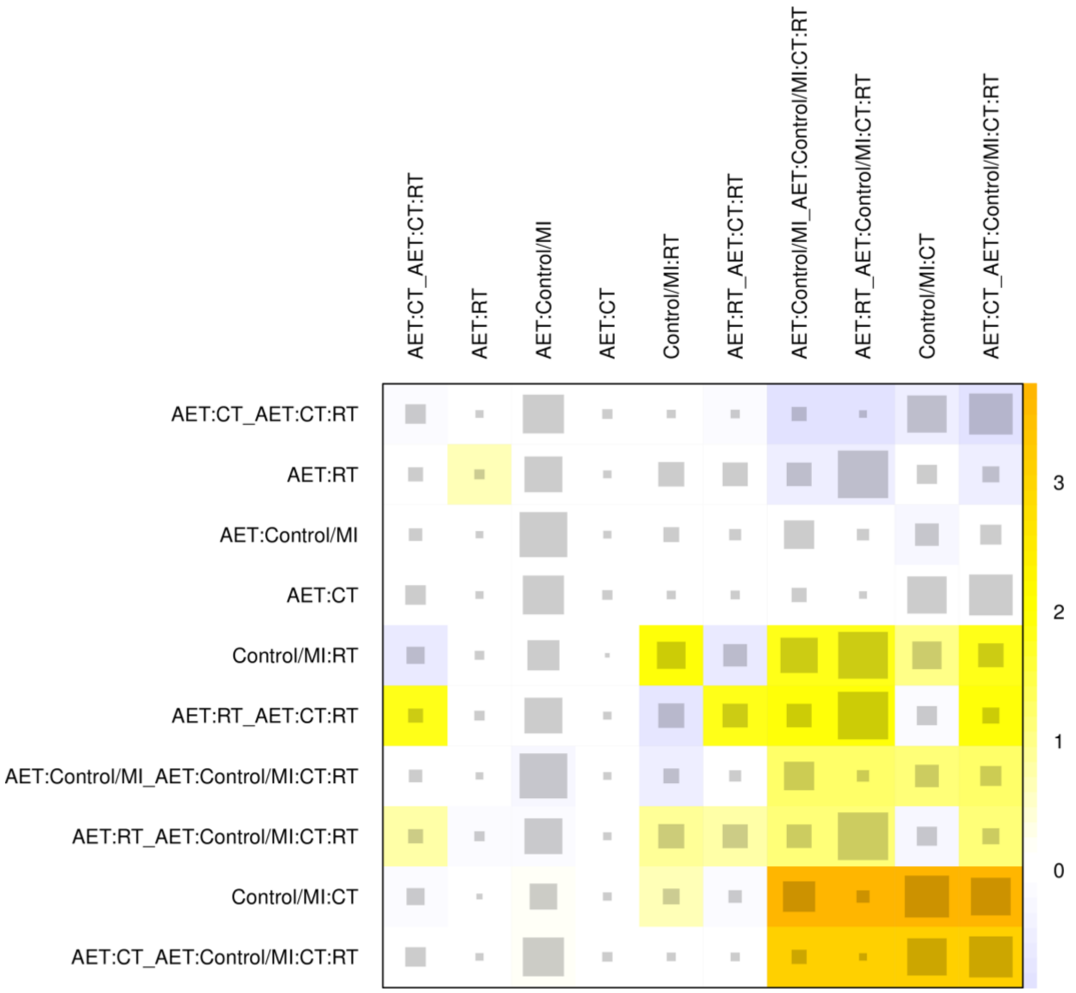

Figure S4. Net-heat plot to assess inconsistency for body mass index.

*This plot is a heat map where the colours on the diagonal represent the inconsistency contribution of the corresponding design and the colours on the off-diagonal are associated with the change in inconsistency between direct and indirect evidence in a network estimate in the row after relaxing the consistency assumption for the effect of a design in the column. A blue coloured element indicates that the evidence of the design in the column supports the evidence in the row; a red coloured element indicates that the evidence of the design in the column contrasts to the evidence in the row.

Online Supporting Information

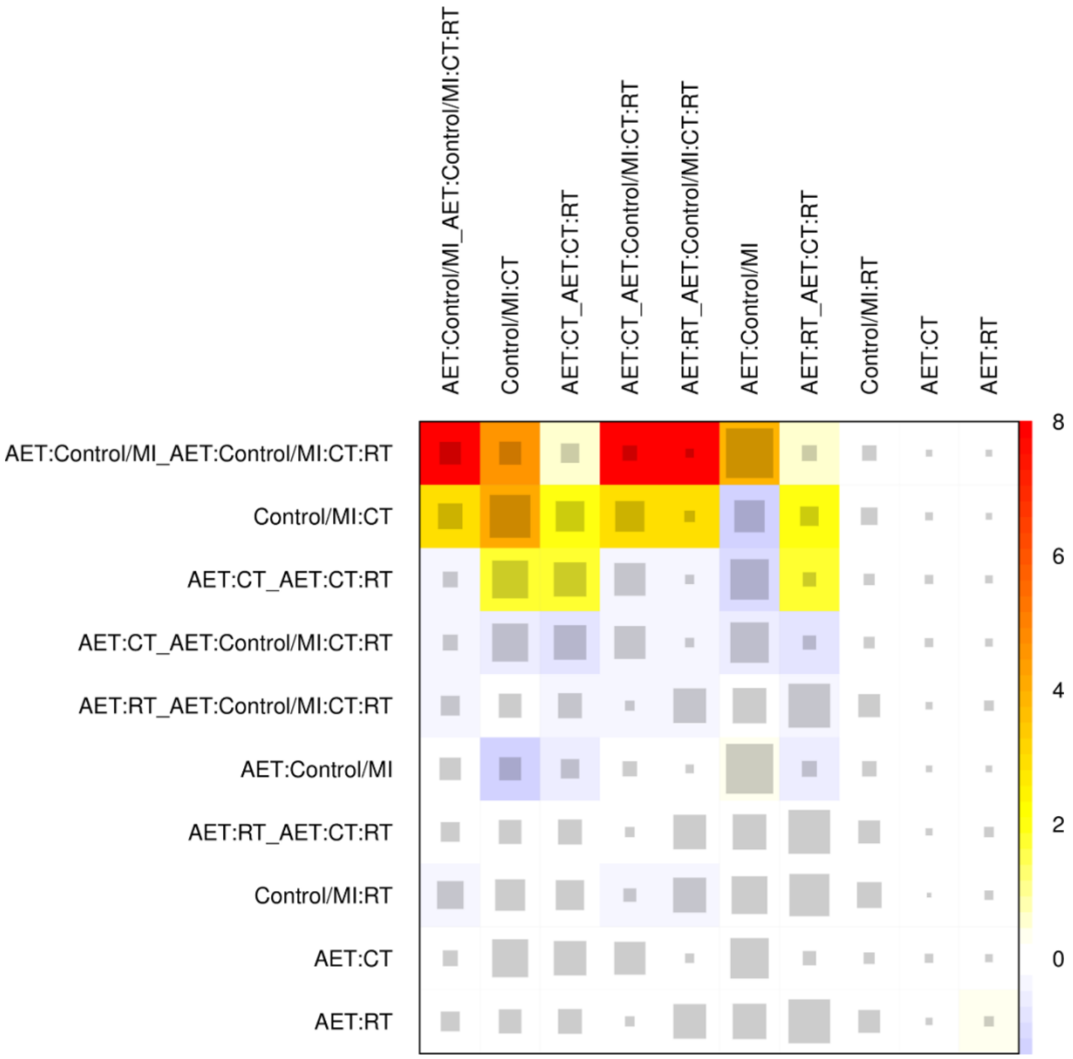

Figure S5. Net-heat plot to assess inconsistency for waist circumference.

*This plot is a heat map where the colours on the diagonal represent the inconsistency contribution of the corresponding design and the colours on the off-diagonal are associated with the change in inconsistency between direct and indirect evidence in a network estimate in the row after relaxing the consistency assumption for the effect of a design in the column. A blue coloured element indicates that the evidence of the design in the column supports the evidence in the row; a red coloured element indicates that the evidence of the design in the column contrasts to the evidence in the row.

Online Supporting Information

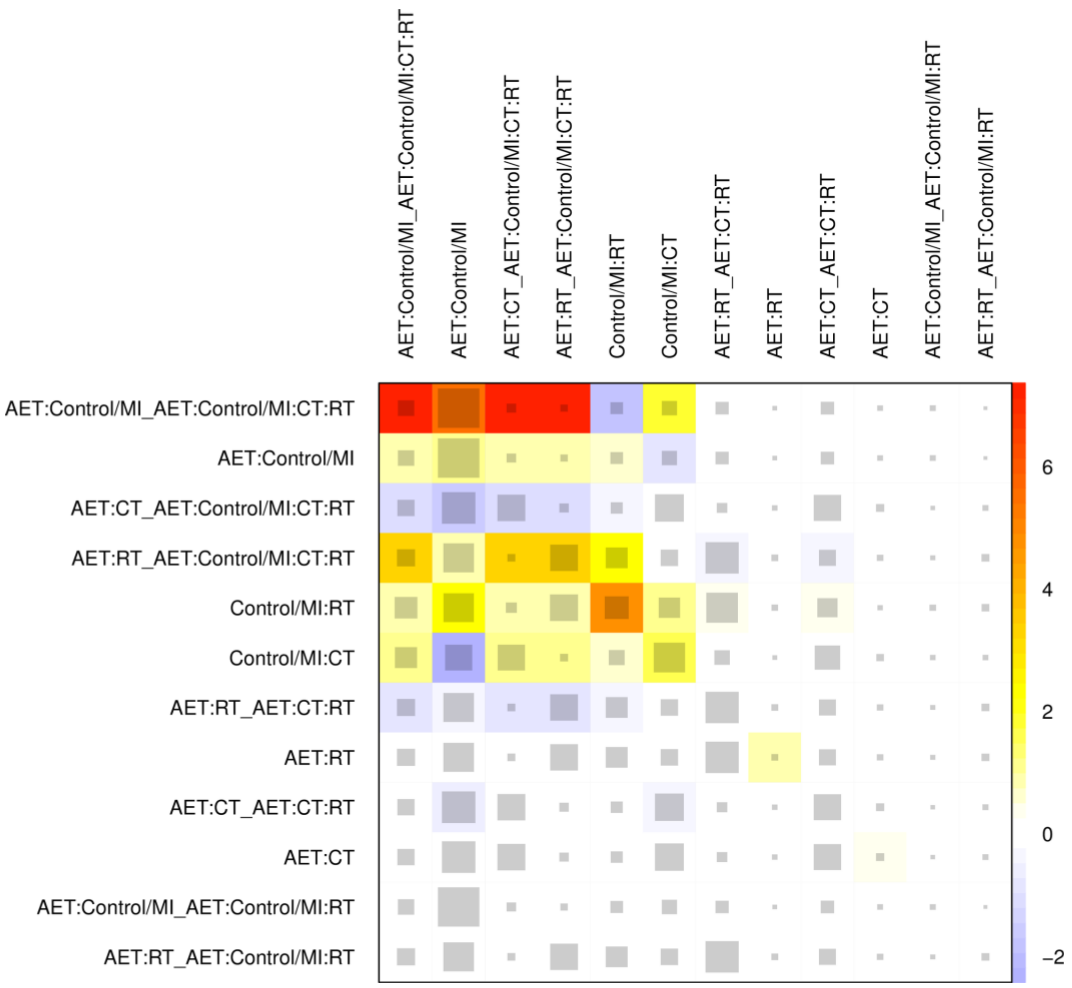

Figure S6. Net-heat plot to assess inconsistency for fat mass.

*This plot is a heat map where the colours on the diagonal represent the inconsistency contribution of the corresponding design and the colours on the off-diagonal are associated with the change in inconsistency between direct and indirect evidence in a network estimate in the row after relaxing the consistency assumption for the effect of a design in the column. A blue coloured element indicates that the evidence of the design in the column supports the evidence in the row; a red coloured element indicates that the evidence of the design in the column contrasts to the evidence in the row.

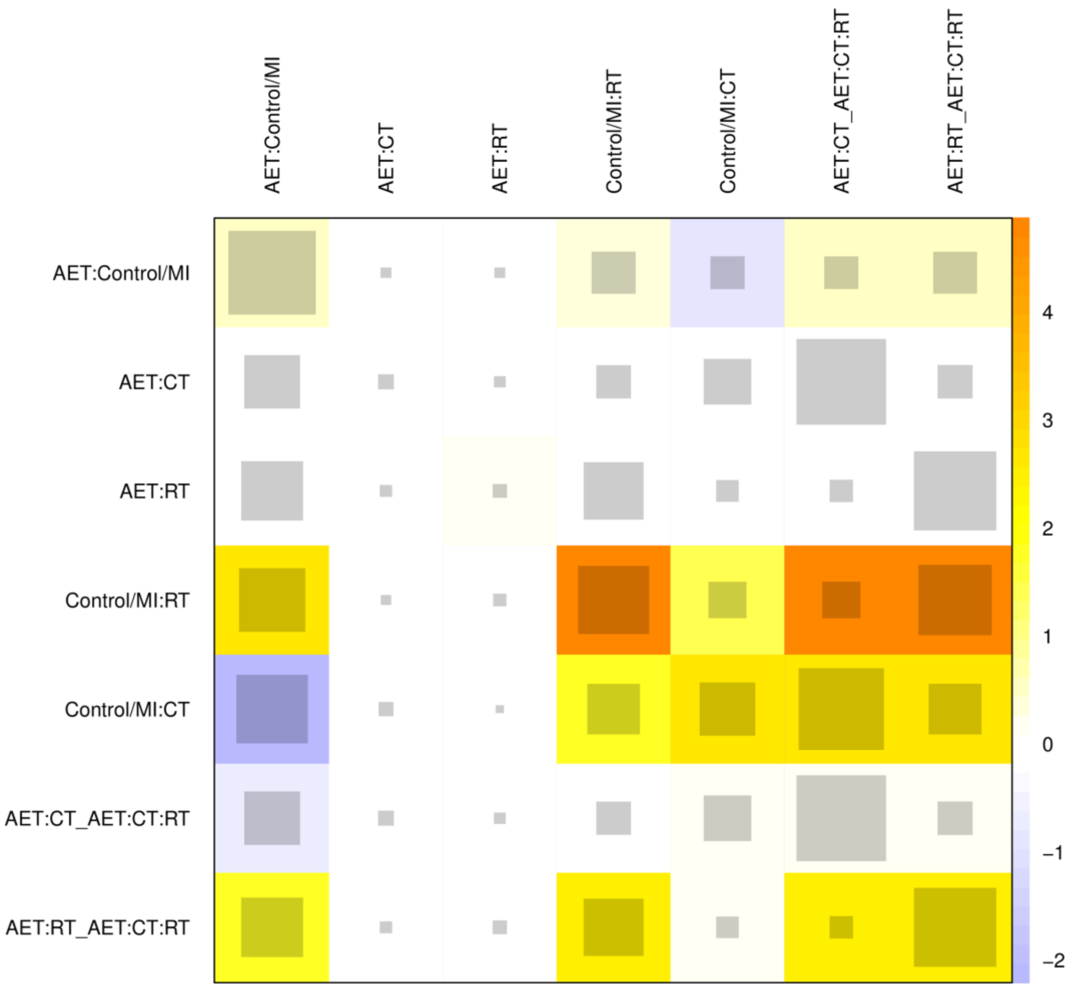

Figure S7. Net-heat plot to assess inconsistency for fat-free mass.

*This plot is a heat map where the colours on the diagonal represent the inconsistency contribution of the corresponding design and the colours on the off-diagonal are associated with the change in inconsistency between direct and indirect evidence in a network estimate in the row after relaxing the consistency assumption for the effect of a design in the column. A blue coloured element indicates that the evidence of the design in the column supports the evidence in the row; a red coloured element indicates that the evidence of the design in the column contrasts to the evidence in the row.

Online Supporting Information

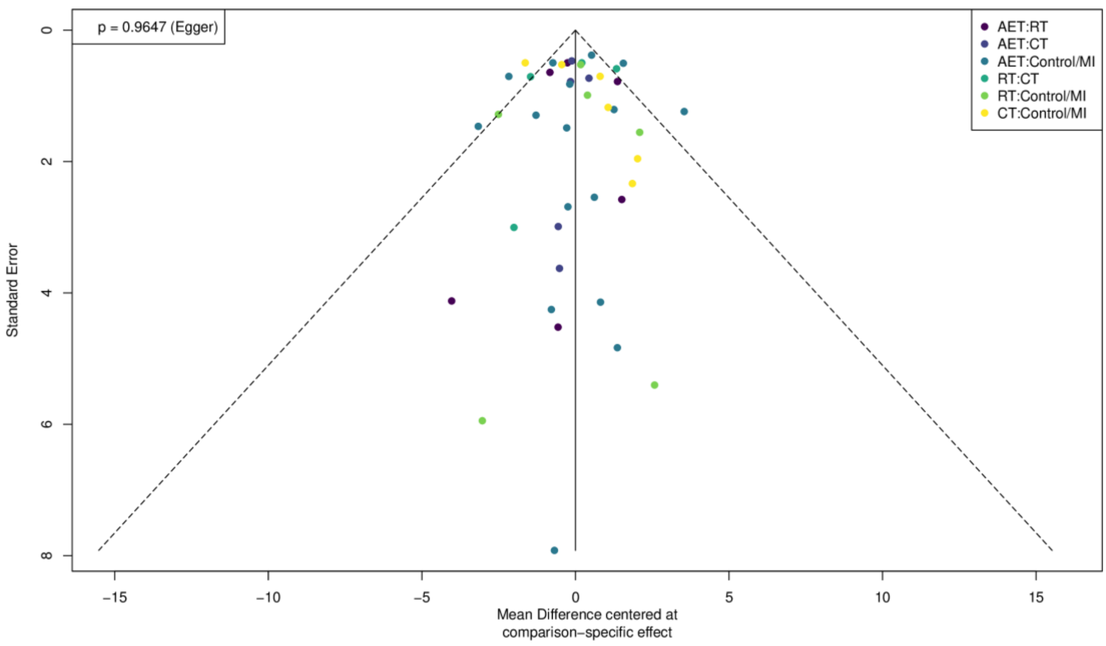

Figure S8. Comparison-adjusted funnel plot for body weight.

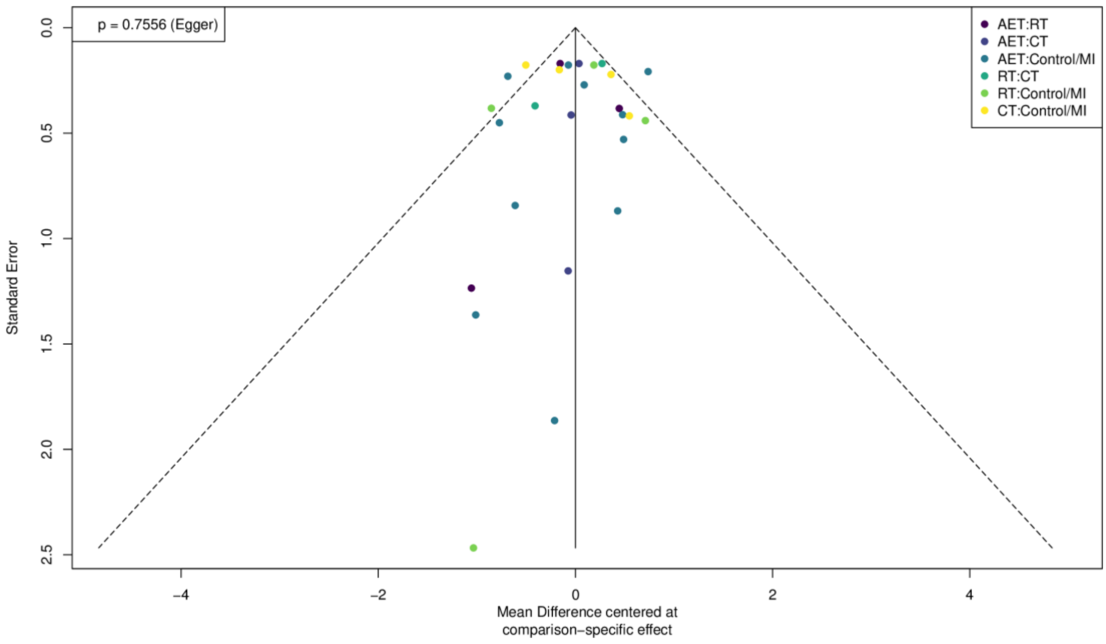

Figure S9. Comparison-adjusted funnel plot for body weight.

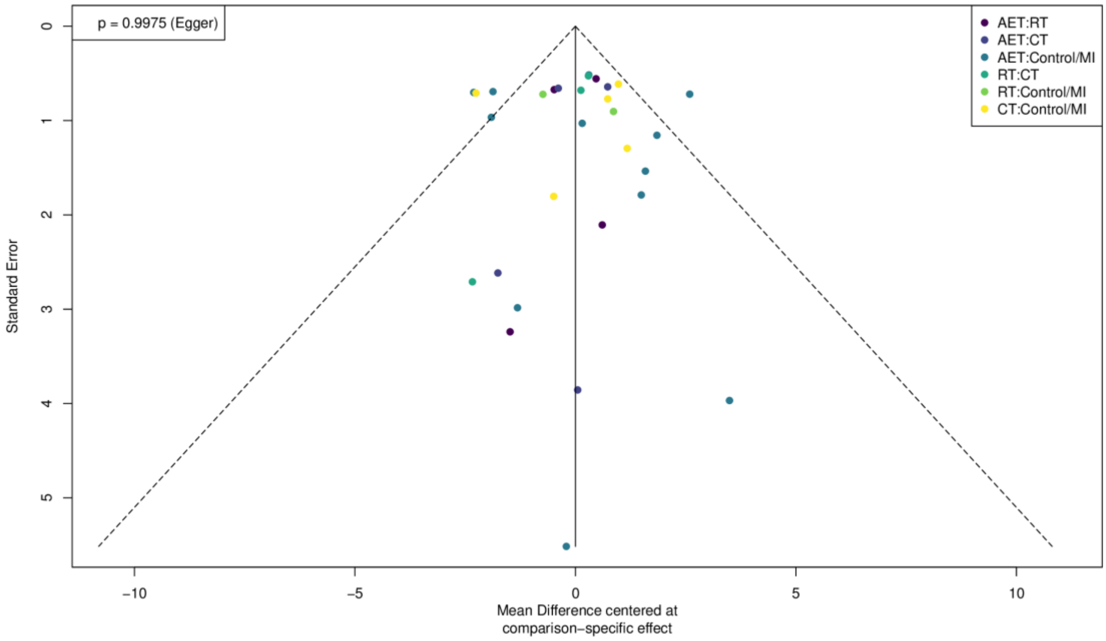

Figure S10. Comparison-adjusted funnel plot for waist circumference.

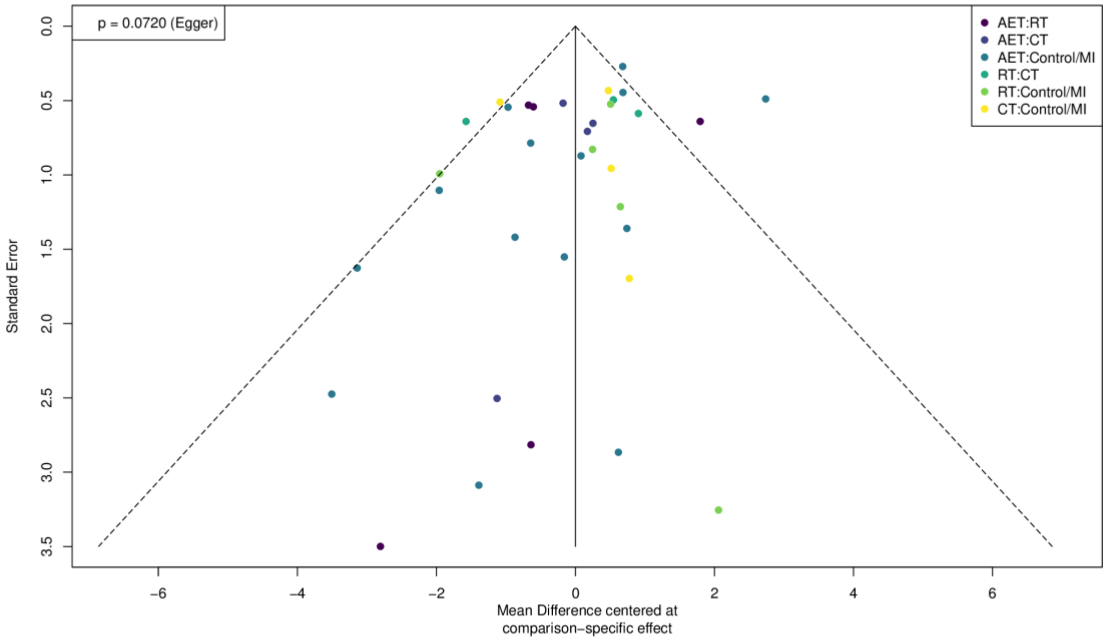

Figure S11. Comparison-adjusted funnel plot for fat mass.

Online Supporting Information

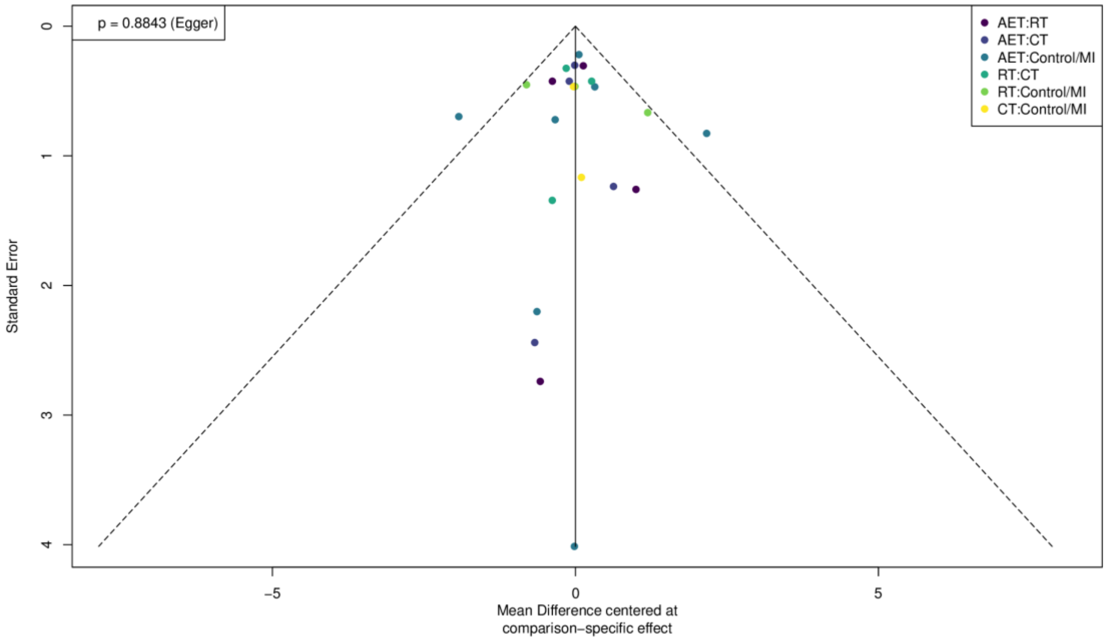

Figure S12. Comparison-adjusted funnel plot for fat-free mass.

Table S13. Subgroup analysis presenting mean differences* with 95% CI in body weight between different training modalities, stratified by mean age: <65 years (bottom-left) and ≥65 years (upper-right).

AET	-1.21 [-2.86; 0.45]	-0.96 [-2.60; 0.69]	**-2.09 [-3.80; -0.38]**
-2.43 [-4.02; -0.84]	RT	0.25 [-1.29; 1.80]	-0.88 [-2.39; 0.62]
-0.79 [-2.19; 0.61]	1.64 [-0.07; 3.35]	CT	-1.14 [-2.60; 0.32]
**-2.18 [-3.00; -1.35]**	**-1.39 [-2.69; -0.08]**	0.26 [-1.32; 1.83]	Control/MI

*Negative value of mean difference favours training modality to the left, whereas positive the one to the right.

AET – aerobic exercise training, CT – combined training, MI – minimal intervention, RT – resistance training;

Table S14. Subgroup analysis presenting mean differences with 95% CI in body mass index between different training modalities, stratified by mean age: <65 years (bottom-left) and ≥65 years (upper-right).

AET	-0.53 [-1.15; 0.09]	-0.25 [-0.88; 0.38]	**-0.72 [-1.39; -0.04]**
0.05 [-1.09; 1.19]	RT	0.28 [-0.31; 0.86]	-0.19 [-0.78; 0.41]
-0.64 [-1.45; 0.18]	-0.69 [-1.98; 0.61]	CT	-0.47 [-1.06; 0.13]
**-1.02 [-1.45; -0.58]**	-1.07 [-2.15; 0.02]	-0.38 [-1.09; 0.34]	Control/MI

Table S15. Subgroup analysis presenting mean differences with 95% CI in waist circumference between different training modalities, stratified by mean age: <65 years (bottom-left) and ≥65 years (upper-right).

AET	-1.90 [-5.36; 1.56]	-1.22 [-4.42; 1.99]	**-4.02 [-7.25; -0.80]**
-1.20 [-2.77; 0.36]	RT	0.68 [-2.54; 3.91]	-2.12 [-5.36; 1.12]
-0.37 [-1.80; 1.05]	0.83 [-0.87; 2.53]	CT	**-2.81 [-5.13; -0.49]**
**-2.06 [-2.95; -1.17]**	-0.85 [-2.42; 0.72]	**-1.68 [-3.02; -0.34]**	Control/MI

Table S16. Subgroup analysis presenting mean differences with 95% CI in fat mass between different training modalities, stratified by mean age: <65 years (bottom-left) and ≥65 years (upper-right).

AET	-0.10 [-1.57; 1.36]	0.29 [-1.20; 1.77]	**-1.85 [-3.35; -0.36]**
-1.22 [-2.77; 0.32]	RT	0.39 [-1.00; 1.78]	**-1.75 [-3.06; -0.44]**
0.32 [-1.20; 1.84]	1.55 [-0.24; 3.33]	CT	**-2.14 [-3.48; -0.80]**
**-1.44 [-2.23; -0.66]**	-0.22 [-1.76; 1.32]	**-1.77 [-3.26; -0.27]**	Control/MI

Table S17. Subgroup analysis presenting mean differences with 95% CI in fat-free mass between different training modalities, stratified by mean age: <65 years (bottom-left) and ≥65 years (upper-right).

AET	-1.33 [-3.31; 0.65]	-1.44 [-3.43; 0.55]	-0.28 [-2.35; 1.79]
-0.85 [-1.75; 0.05]	RT	-0.11 [-1.77; 1.55]	1.05 [-0.37; 2.47]
-0.74 [-1.78; 0.31]	0.11 [-0.97; 1.19]	CT	1.16 [-0.34; 2.66]
0.06 [-0.58; 0.69]	0.90 [-0.05; 1.86]	0.79 [-0.38; 1.96]	Control/MI

Table S18. Subgroup analysis presenting mean differences with 95% CI in body weight between different training modalities, stratified by mean BMI: <35 kg/m^2 (bottom-left) and ≥35 kg/m^2 (upper-right).

AET	-0.59 [-2.08; 0.90]	-0.50 [-2.00; 0.99]	-0.21 [-2.43; 2.00]
**-1.88 [-3.12; -0.65]**	RT	0.09 [-1.21; 1.38]	0.37 [-1.57; 2.32]
-0.79 [-1.93; 0.36]	1.10 [-0.22; 2.41]	CT	0.29 [-1.56; 2.14]
**-2.29 [-3.06; -1.52]**	-0.41 [-1.60; 0.78]	**-1.50 [-2.56; -0.45]**	Control/MI

*Negative value of mean difference favours training modality to the left, whereas positive the one to the right.

AET – aerobic exercise training, CT – combined training, MI – minimal intervention, RT – resistance training;

Table S19. Subgroup analysis presenting mean differences with 95% CI in body mass index between different training modalities, stratified by mean BMI: <35 kg/m^2 (bottom-left) and ≥35 kg/m^2 (upper-right).

AET	-0.10 [-0.85; 0.65]	-0.20 [-1.01; 0.61]	0.01 [-1.13; 1.14]
-0.49 [-1.19; 0.20]	RT	-0.10 [-0.83; 0.63]	0.11 [-0.74; 0.96]
-0.48 [-1.05; 0.09]	0.02 [-0.73; 0.76]	CT	0.21 [-0.91; 1.33]
**-0.99 [-1.37; -0.61]**	-0.50 [-1.16; 0.16]	**-0.51 [-1.00; -0.03]**	Control/MI

Table S20. Subgroup analysis presenting mean differences with 95% CI in waist circumference between different training modalities, stratified by mean BMI: <35 kg/m^2 (bottom-left) and ≥35 kg/m^2 (upper-right).

AET	NA	1.66 [-2.98; 6.30]	-0.91 [-3.92; 2.10]
-1.23 [-2.61; 0.14]	RT	NA	NA
-0.38 [-1.64; 0.87]	0.85 [-0.62; 2.31]	CT	-2.57 [-6.10; 0.97]
**-2.40 [-3.30; -1.49]**	-1.16 [-2.54; 0.21]	**-2.01 [-3.17; -0.85]**	Control/MI

Table S21. Subgroup analysis presenting mean differences with 95% CI in fat mass between different training modalities, stratified by mean BMI: <35 kg/m2 (bottom-left) and ≥35 kg/m2 (upper-right).

AET	0.85 [-0.35; 2.06]	0.69 [-0.63; 2.02]	-0.35 [-2.14; 1.44]
-0.74 [-1.96; 0.47]	RT	-0.16 [-1.31; 0.99]	-1.20 [-2.78; 0.37]
0.46 [-0.76; 1.68]	1.20 [-0.17; 2.57]	CT	-1.04 [-2.54; 0.45]
**-1.66 [-2.41; -0.92]**	-0.92 [-2.09; 0.25]	**-2.12 [-3.29; -0.95]**	Control/MI

Table S22. Subgroup analysis presenting mean differences with 95% CI in fat-free mass between different training modalities, stratified by mean BMI: <35 kg/m^2 (bottom-left) and ≥35 kg/m^2 (upper-right).

AET	**-1.63 [-2.44; -0.82]**	**-1.07 [-1.88; -0.26]**	0.43 [-0.62; 1.47]
-0.59 [-1.44; 0.27]	RT	0.56 [-0.18; 1.29]	**2.05 [1.16; 2.95]**
-0.78 [-1.80; 0.24]	-0.19 [-1.26; 0.87]	CT	**1.50 [0.71; 2.28]**
-0.01 [-0.64; 0.63]	0.58 [-0.24; 1.40]	0.77 [-0.30; 1.85]	Control/MI

Table S23. Subgroup analysis presenting mean differences with 95% CI in body weight between different training modalities, stratified by length of follow-up: <12 months (bottom-left) and ≥12 months (upper-right).

AET	1.77 [-0.78; 4.31]	**-1.10 [-2.12; -0.08]**	**-1.86 [-2.53; -1.18]**
**-1.94 [-3.09; -0.80]**	RT	**-2.86 [-5.43; -0.30]**	**-3.62 [-6.08; -1.17]**
-0.49 [-1.73; 0.76]	**1.46 [0.19; 2.73]**	CT	**-0.76 [-1.52; 0.00]**
**-2.33 [-3.25; -1.41]**	-0.39 [-1.60; 0.82]	**-1.85 [-3.19; -0.50]**	Control/MI

*Negative value of mean difference favours training modality to the left, whereas positive the one to the right.

AET – aerobic exercise training, CT – combined training, MI – minimal intervention, RT – resistance training;

Table S24. Subgroup analysis presenting mean differences with 95% CI in body mass index between different training modalities, stratified by length of follow-up: <12 months (bottom-left) and ≥12 months (upper-right).

AET	-0.60 [-1.94; 0.74]	-0.24 [-1.26; 0.77]	-0.51 [-1.44; 0.42]
-0.36 [-0.95; 0.23]	RT	0.36 [-0.68; 1.40]	0.09 [-0.87; 1.06]
-0.14 [-0.79; 0.51]	0.22 [-0.47; 0.91]	CT	-0.27 [-0.67; 0.13]
**-1.10 [-1.49; -0.70]**	**-0.74 [-1.34; -0.14]**	**-0.95 [-1.64; -0.27]**	Control/MI

Table S25. Subgroup analysis presenting mean differences with 95% CI in waist circumference between different training modalities, stratified by length of follow-up: <12 months (bottom-left) and ≥12 months (upper-right).

AET	NA	-0.94 [-2.22; 0.35]	**-2.22 [-3.12; -1.32]**
-1.11 [-2.66; 0.43]	RT	NA	NA
0.01 [-1.61; 1.63]	1.12 [-0.62; 2.86]	CT	**-1.28 [-2.19; -0.38]**
**-2.44 [-3.55; -1.32]**	-1.32 [-2.92; 0.27]	**-2.44 [-4.11; -0.78]**	Control/MI

Table S26. Subgroup analysis presenting mean differences with 95% CI in fat mass between different training modalities, stratified by length of follow-up: <12 months (bottom-left) and ≥12 months (upper-right).

AET	2.09 [-0.30; 4.47]	-0.12 [-1.57; 1.33]	**-1.41 [-2.31; -0.52]**
-0.67 [-1.84; 0.51]	RT	-2.21 [-4.70; 0.27]	**-3.50 [-5.71; -1.29]**
0.51 [-0.77; 1.80]	1.18 [-0.15; 2.52]	CT	**-1.29 [-2.43; -0.15]**
**-1.54 [-2.48; -0.60]**	-0.87 [-2.08; 0.34]	-2.06 [-3.42; -0.69]	Control/MI

Table S27. Subgroup analysis presenting mean differences with 95% CI in fat-free mass between different training modalities, stratified by length of follow-up: <12 months (bottom-left) and ≥12 months (upper-right).

**AET**	0.71 [-0.55; 1.97]	-1.26 [-2.54; 0.02]	0.41 [-0.48; 1.31]
**-1.23 [-1.87; -0.58]**	RT	**-1.97 [-3.24; -0.70]**	**-0.30 [-1.18; 0.58]**
**-0.92 [-1.62; -0.21]**	0.31 [-0.42; 1.04]	CT	1.67 [0.76; 2.58]
0.00 [-0.59; 0.60]	**0.92 [0.09; 1.75]**	**1.23 [0.53; 1.93]**	Control/MI

Table S28. Subgroup analysis presenting mean differences with 95% CI in body weight between different training modalities, stratified by use of dietary co-intervention: absent (bottom-left) and present (upper-right).

AET	-0.87 [-2.01; 0.28]	**-1.36 [-2.48; -0.23]**	**-1.80 [-2.82; -0.78]**
**-2.31 [-3.81; -0.81]**	RT	-0.49 [-1.55; 0.57]	-0.93 [-1.92; 0.06]
-0.26 [-1.59; 1.07]	**2.05 [0.50; 3.59]**	CT	-0.44 [-1.34; 0.45]
**-2.29 [-3.19; -1.39]**	0.02 [-1.54; 1.58]	**2.03 [-3.35; -0.70]**	Control/MI

*Negative value of mean difference favours training modality to the left, whereas positive the one to the right.

AET – aerobic exercise training, CT – combined training, MI – minimal intervention, RT – resistance training;

Table S29. Subgroup analysis presenting mean differences with 95% CI in body mass index between different training modalities, stratified by use of dietary co-intervention: absent (bottom-left) and present (upper-right).

AET	-0.11 [-0.83; 0.61]	-0.46 [-1.19; 0.28]	-0.59 [-1.25; 0.07]
-0.74 [-1.51; 0.03]	RT	-0.35 [-1.02; 0.32]	-0.49 [-1.06; 0.09]
-0.25 [-0.87; 0.37]	0.49 [-0.32; 1.30]	CT	-0.14 [-0.67; 0.40]
**-1.04 [-1.43; -0.66]**	-0.31 [-1.07; 0.45]	**-0.79 [-1.37; -0.22]**	Control/MI

Table S30. Subgroup analysis presenting mean differences with 95% CI in waist circumference between different training modalities, stratified by use of dietary co-intervention: absent (bottom-left) and present (upper-right).

AET	-0.96 [-2.98; 1.06]	-0.77 [-2.77; 1.22]	**-2.21 [-3.77; -0.65]**
-1.18 [-3.07; 0.72]	RT	0.19 [-1.83; 2.21]	-1.25 [-2.83; 0.34]
-0.14 [-1.73; 1.44]	1.03 [-0.90; 2.97]	CT	**-1.44 [-2.77; -0.10]**
**-2.36 [-3.46; -1.25]**	-1.18 [-3.14; 0.78]	**-2.21 [-3.74; -0.68]**	Control/MI

Table S31. Subgroup analysis presenting mean differences with 95% CI in fat mass between different training modalities, stratified by use of dietary co-intervention: absent (bottom-left) and present (upper-right).

AET	0.41 [-0.53; 1.35]	0.08 [-1.03; 1.19]	**-1.63 [-2.46; -0.79]**
-1.24 [-2.66; 0.17]	RT	-0.33 [-1.38; 0.73]	**-2.04 [-2.89; -1.19]**
0.55 [-0.70; 1.79]	**1.79 [0.32; 3.26]**	CT	**-1.71 [-2.77; -0.66]**
**-1.32 [-2.19; -0.45]**	-0.07 [-1.55; 1.40]	**-1.86 [-3.10; -0.63]**	Control/MI

Table S32. Subgroup analysis presenting mean differences with 95% CI in fat-free mass between different training modalities, stratified by use of dietary co-intervention: absent (bottom-left) and present (upper-right).

AET	**-0.93 [-1.86; 0.00]**	**-1.07 [-2.08; -0.06]**	-0.00 [-0.85; 0.84]
-1.25 [-3.73; 1.23]	RT	-0.14 [-1.12; 0.84]	**0.92 [0.18; 1.67]**
-1.03 [-3.32; 1.26]	0.22 [-2.27; 2.71]	CT	**1.06 [0.15; 1.98]**
0.17 [-1.38; 1.72]	1.20 [-1.57; 3.97]	1.42 [-1.50; 4.35]	Control/MI

Table S33. Sensitivity analysis presenting mean differences with 95% CI in body weight between different training modalities, after exclusion of studies with overall high risk of bias.

AET			
**-1.59 [-2.94; -0.25]**	RT		
**-1.11 [-2.21; -0.00]**	0.49 [-0.89; 1.87]	CT	
**-2.08 [-2.86; -1.29]**	-0.48 [-1.82; 0.85]	-0.97 [-1.96; 0.02]	Control/MI

*Negative value of mean difference favours training modality to the left, whereas positive the one to the right.

AET – aerobic exercise training, CT – combined training, MI – minimal intervention, RT – resistance training;

Table S34. Sensitivity analysis presenting mean differences with 95% CI in body mass index between different training modalities, after exclusion of studies with overall high risk of bias.

AET			
**-0.62 [-1.19; -0.05]**	RT		
-0.42 [-0.90; 0.07]	0.21 [-0.37; 0.78]	CT	
**-0.87 [-1.24; -0.51]**	-0.25 [-0.80; 0.29]	**-0.46 [-0.88; -0.04]**	Control/MI

Table S35. Sensitivity analysis presenting mean differences with 95% CI in weight circumference between different training modalities, after exclusion of studies with overall high risk of bias.

AET			
-1.03 [-3.32; 1.25]	RT		
-0.65 [-2.26; 0.96]	0.38 [-1.98; 2.75]	CT	
**-2.49 [-3.59; -1.38]**	-1.45 [-3.73; 0.83]	**-1.83 [-3.22; -0.45]**	Control/MI

Table S36. Sensitivity analysis presenting mean differences with 95% CI in fat mass between different training modalities, after exclusion of studies with overall high risk of bias.

AET			
-0.30 [-1.63; 1.03]	RT		
0.28 [-0.87; 1.42]	0.57 [-0.82; 1.97]	CT	
**-1.38 [-2.13; -0.62]**	**-1.08 [-2.39; 0.24]**	**-1.65 [-2.72; -0.58]**	Control/MI

Table S37. Sensitivity analysis presenting mean differences with 95% CI in fat-free mass between different training modalities, after exclusion of studies with overall high risk of bias.

AET			
**-1.53 [-2.50; -0.56]**	RT		
**-1.13 [-2.01; -0.24]**	0.40 [-0.62; 1.43]	CT	
0.27 [-0.36; 0.91]	**1.80 [0.82; 2.78]**	**1.40 [0.54; 2.27]**	Control/MI

Table S38. Sensitivity analysis presenting mean differences with 95% CI in body weight between different training modalities, after splitting control and minimal intervention into separate nodes.

AET				
**-1.76 [-2.77; -0.75]**	RT			
-0.76 [-1.72; 0.21]	1.00 [-0.07; 2.08]	CT		
-1.26 [-2.76; 0.24]	0.50 [-1.18; 2.18]	-0.50 [-1.97; 0.96]	MI	
**-2.36 [-3.11; -1.61]**	-0.60 [-1.61; 0.40]	**-1.61 [-2.58; -0.63]**	-1.10 [-2.69; 0.48]	Control

*Negative value of mean difference favours training modality to the left, whereas positive the one to the right.

AET – aerobic exercise training, CT – combined training, MI – minimal intervention, RT – resistance training;

Table S39. Sensitivity analysis presenting mean differences with 95% CI in body mass index between different training modalities, after splitting control and minimal intervention into separate nodes.

AET				
**-0.52 [-1.02; -0.02]**	RT			
-0.33 [-0.80; 0.14]	0.19 [-0.36; 0.73]	CT		
-0.51 [-1.12; 0.10]	0.01 [-0.71; 0.73]	-0.18 [-0.74; 0.39]	MI	
**1.05 [-1.41; -0.70]**	**-0.53 [-1.01; -0.06]**	**-0.72 [-1.19; -0.25]**	-0.54 [-1.19; 0.11]	Control

Table S40. Sensitivity analysis presenting mean differences with 95% CI in weight circumference between different training modalities, after splitting control and minimal intervention into separate nodes.

AET				
**-1.25 [-2.34; -0.15]**	RT			
-0.09 [-1.10; 0.91]	1.15 [-0.01; 2.32]	CT		
**-0.59 [-2.12; 0.93]**	0.66 [-1.10; 2.41]	-0.50 [-2.00; 1.00]	MI	
**-2.70 [-3.46; -1.94]**	**-1.45 [-2.57; -0.34]**	**-2.61 [-3.60; -1.61]**	**-2.11 [-3.72; -0.50]**	Control

Table S41. Sensitivity analysis presenting mean differences with 95% CI in fat mass between different training modalities, after splitting control and minimal intervention into separate nodes.

AET				
-0.47 [-1.49; 0.56]	RT			
0.45 [-0.60; 1.50]	0.92 [-0.24; 2.07]	CT		
-0.85 [-2.49; 0.79]	-0.39 [-2.21; 1.44]	-1.30 [-2.94; 0.34]	MI	
**-1.69 [-2.45; -0.93]**	**-1.22 [-2.24; -0.21]**	**-2.14 [-3.23; -1.05]**	-0.84 [-2.57; 0.90]	Control

Table S42. Sensitivity analysis presenting mean differences with 95% CI in fat-free mass between different training modalities, after splitting control and minimal intervention into separate nodes.

AET				
**-1.01 [-1.76; -0.25]**	RT			
**-1.01 [-1.81; -0.21]**	-0.01 [-0.83; 0.82]	CT		
0.19 [-1.13; 1.51]	1.20 [-0.32; 2.72]	1.21 [-0.34; 2.75]	MI	
-0.01 [-0.70; 0.68]	**1.00 [0.28; 1.71]**	**1.00 [0.18; 1.82]**	-0.20 [-1.69; 1.29]	Control

Online Supporting Information

Table S43. Between-study heterogeneity (network τ^2 and I^2 statistic) in subgroup and sensitivity analyses.

**Analysis**	**Body weight**		**Body mass index**		**Waist circumference**		**Fat mass**		**Fat-free mass**	
	τ^2	I^2	τ^2	I^2	τ^2	I^2	τ^2	I^2	τ^2	I^2
**Main (95% CI)**	0.95	52.6% [29.8%; 67.9%]	0.18	62.1% [39.3%; 76.4%]	1.21	56.8% [31.3%; 72.9%]	0.96	63.4% [44.8%; 75.8%]	0.31	50.2% [14.3%; 71.0%]
**Age**										
<65 years	1.03	49.1%	0.25	61.1%	1.07	50.5%	0.98	61.7%	0.31	44.2%
>65 years	1.20	56.4%	0.14	58.2%	2.67	64.7%	0.97	59.8%	1.13	72.5%
**Baseline BMI**										
BMI <35 kg/m²	1.07	57.5%	0.22	67.6%	1.29	60.9%	1.07	67.9%	0.36	48.3%
BMI <35 kg/m²	0.00	0.0%	0.00	0.0%	NA	NA	0.00	0.0%	0.00	0.0%
**Length of follow-up**										
<12 months	1.12	53.2%	0.20	62.0%	1.74	61.0%	1.30	65.5%	0.21	37.4%
>12 months	0.00	0.0%	0.06	36.6%	0.00	0.0%	0.29	31.2%	0.00	0.0%
**Dietary co-intervention**										
No	1.08	67.5%	0.17	64.3%	1.86	70.9%	0.96	73.4%	1.58	72.6%
Yes	0.00	0.0%	0.11	33.5%	0.00	0.0%	0.00	0.0%	0.35	44.7%
**Exclusion of studies with high risk of bias**	1.00	58.0%	0.17	66.4%	1.89	69.2%	0.98	69.2%	0.27	41.5%
**Analysis with 5 nodes**	0.88	48.9%	0.15	56.0%	0.62	39.1%	1.06	64.0%	0.41	51.8%
